# The natural history of molecular functions inferred from an extensive phylogenomic analysis of gene ontology data

**DOI:** 10.1371/journal.pone.0176129

**Published:** 2017-05-03

**Authors:** Ibrahim Koç, Gustavo Caetano-Anollés

**Affiliations:** 1 Molecular Biology and Genetics, Gebze Technical University, Kocaeli, Turkey; 2 Evolutionary Bioinformatics Laboratory, Department of Crop Sciences, University of Illinois at Urbana-Champaign, Urbana, IL, United States of America; University of Georgia, UNITED STATES

## Abstract

The origin and natural history of molecular functions hold the key to the emergence of cellular organization and modern biochemistry. Here we use a genomic census of Gene Ontology (GO) terms to reconstruct phylogenies at the three highest (1, 2 and 3) and the lowest (terminal) levels of the hierarchy of molecular functions, which reflect the broadest and the most specific GO definitions, respectively. These phylogenies define evolutionary timelines of functional innovation. We analyzed 249 free-living organisms comprising the three superkingdoms of life, Archaea, Bacteria, and Eukarya. Phylogenies indicate catalytic, binding and transport functions were the oldest, suggesting a ‘metabolism-first’ origin scenario for biochemistry. Metabolism made use of increasingly complicated organic chemistry. Primordial features of ancient molecular functions and functional recruitments were further distilled by studying the oldest child terms of the oldest level 1 GO definitions. Network analyses showed the existence of an hourglass pattern of enzyme recruitment in the molecular functions of the directed acyclic graph of molecular functions. Older high-level molecular functions were thoroughly recruited at younger lower levels, while very young high-level functions were used throughout the timeline. This pattern repeated in every one of the three mappings, which gave a criss-cross pattern. The timelines and their mappings were remarkable. They revealed the progressive evolutionary development of functional toolkits, starting with the early rise of metabolic activities, followed chronologically by the rise of macromolecular biosynthesis, the establishment of controlled interactions with the environment and self, adaptation to oxygen, and enzyme coordinated regulation, and ending with the rise of structural and cellular complexity. This historical account holds important clues for dissection of the emergence of biomcomplexity and life.

## Introduction

Evolutionary genomics traces phylogenomic relationships between organisms using a range of molecular features, including the sequences, structures and molecular functions of proteins and nucleic acids. Cellular organisms can be grouped into three domains of life, superkingdoms Archaea, Bacteria, and Eukarya. These three supergroups are widely accepted. However, establishing which superkingdom is the most ancient is still a matter of debate. Initially, phylogenetic trees rooted with paralogous gene sequences suggested Archaea and Eukarya were sister groups that diverged from a microbial common ancestor [1–4]. This canonical view positioned Bacteria at the base of the Tree of Life (ToL). However, the methodology was shown to produce discordant topologies depending on the sequences of the selected paralogous gene couples and was prone to technical artifacts [5–7]. Consequently, despite the promises of evolutionary genomics, a ToL potraying the phylogenetic relationships of all superkingdoms and their history remain controversial in evolutionary biology [7]. Phylogenetic trees have been predominantly built from molecular sequences. However, these trees can only be rooted when incorporating a reference taxon in the analysis, i.e. an additional external hypothesis of relationship (outgroup), which is not available for the ToL. Moreover, sequences cannot be reliably used to explore deep phylogenetic relationships [8]. Mutation, recombination, gene duplication and other events of change in protein and nucleic acid sequences occur at fast pace [8–11]. These processes are highly dynamic, can lead to paralogy and mutational saturation, and can restrict accurate phylogenetic analysis to low taxonomy levels [8]. Since sequences provide snapshots of recent evolutionary history, orthologous genes are useful for recontructing phylogeny of closely related organisms. In contrast, and in addition to molecular sequences, protein structural domains [12, 13], domain interactomes [14], metabolic information [15, 16], and ontological terms describing molecular functions [7, 8, 17] have been used to build deeper and global phylogenies of the living world.

The Gene Ontology (GO) database standardizes the functional annotation of gene products with a vocabulary of ontological terms describing the biological processes (bp), molecular functions (mf), and cellular components (cc) of the cell [18, 19]. Molecular functions are often considered activities, actions characterizing agents (also known as actors) of the molecular realm, the gene products. According to philosophical ontology, they are observables that are actively occurring (“occurrents”) and have a beginning and an end. In contrast, cellular components are entities that persist and endure through time (“continuants”), even though they can change (e.g. chromosome, mitochondria). Biological processes are somehow between the mf and cc extremes. They are collectives of events, objects and their properties manifesting in time, behaving as occurents but sometime describing ‘functionings’ as continuants. The relationship of GO terms is complex. GO terms induce a tree-like network structure that is not unifyingly hierarchical. Each of the root terms for bp, mf and cc networks unfolds an independent directed acyclic graph (DAG) where child terms can be connected with multiple parents [8]. GO terms at lower levels (child) of the DAG represent more specialized functional annotations (e.g., “ATP binding” [GO:0005524]) while higher-level terms represent broader functional categories (e.g., “binding” [GO:0005488]) [20, 21]. GO annotations provide a very comprehensive and controlled vocabulary that is also very informative at evolutionary levels [8, 21]. While GO terms are semantic explanations of gene products that by ontological definition lack evolutionary meaning, it is generally considered that ancient molecules served multiple functions and showed broad specificity and that these molecular functions diversified into more specific and efficient counterparts during evolution, leading to the extraordinarily diverse and specific functions that exist in the modern biological world [17, 22–25]. The assumption that ancient enzymes were generalist multi-tasking proteins has been borne out thanks to protein resurrection experiments that use phylogenetic reconstruction to design ancestral sequences and synthesize the corresponding proteins [26]. Examples include the reconstruction of an ancestral α-glycosidase that existed ~120 million years ago [27] and ancient bacterial β-lactamases that existed ~3 billion years (Gyr) ago, which showed that the resurrected enzymes were superior generalists capable of catalysis of a broad range of alternative substrates [28]. In particular, the evolutionary increases of specificity in β-lactamases appeared determined by corresponding changes in the conformational dynamics of the molecules [29]. Thus, promiscuous functions can serve as evolutionary starting points to a host of more specialized functions. This notion can be used as rationale for the existence of an intrinsic link between terms of the GO hierarchy and the evolving structure and dynamics of protein molecules, in which higher level GO terms are generally considered analogous to functional forms of more ancient generalist molecules and more specific lower level GO terms are generally considered more modern forms [17].

We note that the GO database annotates extant molecular machinery by assigning GO terminal terms to genes. Thus, higher level GO terms and DAGs built above these terms still represent annotations to present day proteins. However, the implicit link between GO hierarchy and evolution, which manifests in the genomic census of GO terms (occurrents and continuants), can still help interpret phylogenetic signal that is present in the genomic census of vocabulary abstractions of the GO hierarchy. In fact, GO terms in sequenced genomes represent excellent phylogenetic characters that carry considerable phylogenetic signal [8, 17, 21]. When genomes are considered taxa, the tree-building (reconstruction) exercise that minimizes homoplasy (multiple origins) produces phylogenetic trees of organisms, including ToLs. These trees describe the evolution of ‘functionomes’, entire repertoires of bp, mf and cc terms defining the entire functional toolkit of individual organisms. We recently used mf terms defined by the GO database as molecular characters to distinguish cellular species that have been fully sequenced [7, 8, 17, 21]. The repertoire of GO terms that is used as phylogenetic characters portrays the physiologies of the organisms that are being studied. These terms are embedded in molecular structures, which are more conserved than gene sequences and are defined with a controlled vocabulary that is absent in sequences [17, 30, 31]. GO terms therefore provide useful information about species diversification and allow deep evolutionary comparisons. One important limitation associated with this approach is the possible exchange of genetic materials between species, especially akaryotes (i.e Bacteria and Archaea) via horizontal gene transfer (HGT) [7, 32, 33]. Because GO terms are hierarchically structured in the DAG, one terminal GO term of molecular function (GO_TMF_) can be linked to multiple parents. This results in many GO terms having many-to-many relationships being prone to convergent evolution since they have multiple parents [8, 21]. This complicates ToL reconstruction. On the other hand, the resulting hierarchical link can specify patterns of evolutionary diversification that are useful. For example, ToLs reconstructed using molecular functions contributed new ways to rooting phylogenies [7, 8, 17, 21]. These studies suggested termophilic archaeal species were the most ancient form of life [21] and revealed a strong evolutionary association between Bacteria and Eukarya [7]. Furthermore, functionome diversity analyses revealed that ancient catalysts were crucial for binding and transport and, remarkably, that the majority of novel functions appeared late in evolution [17].

In numerous studies, we used the 3-dimensional (3D) structural designs of proteins (their folds) to explore the diversification of organisms (e.g. [13, 30]). The ToLs that were reconstructed were congruent with those built from ontological data. Protein structures hold molecular functions and are therefore more conserved in distant species [13, 17, 30]. They are also robust against the effects of mutation, recombination, cooption and loss. As with molecular functions, protein structures encoded in a genome can be defined at different levels of structural abstraction, including the wide-encompassing fold (F) and fold superfamily (FSF) levels of the Structural Classification of Proteins (SCOP) [34]. While FSFs can be considered evolutionary units, Fs group FSFs with secondary structures that are similarly packed in 3D space but that are not necessarily evolutionary related [30]. The census of protein structural domains in proteomes at these highly conserved levels allow the reconstruction of phylogenetic trees of structural domains [13, 14, 30, 35–37]. These trees are built from the occurrence and abundance of domains in proteomes, which are used as phylogenetic characters in tree reconstructions. The trees describe the evolution of structural domains and can be used to build chronologies of their evolutionary appearance, turning rooted tree visualizations into linear chronological structures.

Since the history of protein domains delimit through their structures the emergence and evolutionary origin of molecular functions [17], here we apply the same strategy of building timelines of domains to the study of the origin and evolution of molecular functions. First, we retrieve mf terms of the GO database that are present in 249 genomes representing all superkingdoms of life. The functional dataset that we assembled explains genomic occurrence and abundance of GO ontology terms. Second, we explore evolutionary relationships of functional terms and categories at different level of the DAG of molecular functions (DAG_mf_) by building phylogenetic trees of functions (ToFs) with homoplasy-minimizing methods. Third, we investigate evolutionary patterns of molecular functions within or among functional categories to reveal the genomic and evolutionary contexts of functional diversification. In our analyses, GO terms are by definition more conserved than the encoding sequences and sometimes more conserved that their associated structures, as previously shown for example by equating a GO level 6 definition of ‘hydrolase activity’ to the P-loop hydrolase fold (see Table S9 in [17]). Loss of molecular functions in a genome can be costly when it involves loss of many genes that have appeared over long periods of evolutionary time [7]. The consequence is that ancient functions have more time to spread in genomes and increase their abundance when compared to those that have appeared recently. Thus, ancient and abundant functions are much more conserved and refractory to the effects of gene gain and loss and HGT, revealing with confidence primordial and very ancient innovations. In previous work, we analyzed the functional dataset of 38 bacterial and eukaryal genomes [17]. Here we extend that initial study to the three superkingdoms using a detailed analysis of more genomes, bringing together heterogeneous information from the genomes of distant species [17, 21] and taking into consideration the limitations of the GO classification [38–40].

## Results

### Phylogenomic analysis of molecular functions at high levels of ontological classification

The GOA project of EBI (http://www.ebi.ac.uk/GOA) provides high-quality electronic and manual annotations for genomes using a GO-based controlled vocabulary. The current analysis revealed 1,891 GO_TMF_ terms in 249 free-living organisms from the three superkingdoms (reproduced from [21]). When we included non-free living organisms and free-living organisms (358 organisms), the ToLs indicated a bias as most of the non-free living organisms appeared at basal positions of the tree [21]. In that analysis, Eukarya did not form a seperate supergroup but derived from a subgroup of Bacteria. Also, non-free living organisms that engage in parasitism have tendencies of genome reduction, which affect the functional metabolic makeup of the genome [41]. To exlcude this bias, we explored the lifestyle of 358 organisms and filtered out 109 organisms with facultative parasitic or obligate parasitic lifestyles. The remaining 249 organisms of our free-living dataset carry the functional reportoire of terminal GO terms of 45 archaeal, 183 bacterial and 21 eukaryal organisms. To better resolve phylogenomic relationships, problematic characters that contribute substantial homoplasy to the data set must be excluded [8, 42]. In particular, HGT is thought to have played an important role in microbial evolution, especially in Bacteria [8, 21]. Since our free-living dataset included a large number of bacterial functionomes (183), the character dataset prone to HGT can add phylogenetic noise and affect results. For that reason, we removed HGT-prone genes from our proteome dataset using HGT-DB [43] by identifying horizontally transferred proteins (HTPs) that were cross-listed in the database and their associated GO terms. When we compared the enrichment of HTPs to the enrichment of remaining terminal GO terms, we found that 115 terminal GO terms were significantly associated with HTPs (P < 0.05). We therefore removed these terms. Their exclusion increased phylogenetic accuracy [8, 17, 21]. Since we already quantified the degree of HGT affecting phylogenetic trees it in several studies [7, 8, 17, 21, 44], we used the non-HGT dataset to reconstruct ToFs describing the evolution of functions from 249 free-living organisms to minimize homoplasy [7, 8, 17, 21, 44].

Table 1 shows general statistics of GO annotations. The number of gene products and their associated terminal terms were 367,598 following removal of ambiguous annotations. In this study, the proportion of gene products annotated with terminal GO terms in a genome (genomic coverage) was at least 50%. We used GO accession numbers to maximize GO terms connected to GO_TMF_ in level 1, level 2, and level 3 of the DAG_mf_ of AmiGO. We used four criteria for choosing taxa, identifying 17 out of 21 molecular functions in 249 organisms at level 1 of molecular function (Table 2). In the case of four remaining level 1 GO terms, all of them did not have any child term (e.g. GO:0036370, GO:0042056, GO:0045499, and GO:0045735).

**Table 1:** General statistics of GO annotations.

Taxon	Archaea	Bacteria	Eukarya
No. genomes	45	183	21
No. GO_mf_ terms	636	1,055	1,629
No. manually annotated genes	892	3,586	48,962
No. genes annotated to terminal GO terms	30,046	223,015	114,537

**Table 2:** List of level 1 GO terms mapped to GO_TMF_ terms, sorted by *nd* values.

GO accession (Level 1)	Definition	Venn group	*nd*
GO:0003824	catalytic activity	ABE	0
GO:0005488	binding	ABE	0.07
GO:0005215	transporter activity	ABE	0.14
GO:0060089	molecular transducer activity	ABE	0.21
GO:0000988	protein binding transcription factor activity	ABE	0.29
GO:0016209	antioxidant activity	ABE	0.36
GO:0009055	electron carrier activity	ABE	0.43
GO:0030234	enzyme regulator activity	ABE	0.5
GO:0005198	structural molecule activity	BE	0.57
GO:0004872	receptor activity	BE	0.64
GO:0001071	nucleic acid binding transcription factor activity	BE	0.71
GO:0016247	channel regulator activity	BE	0.86
GO:0045182	translation regulator activity	E	0.86
GO:0030545	receptor regulator activity	E	0.86
GO:0031386	protein tag	E	0.93
GO:0016530	metallochaperone activity	E	1
GO:0016015	morphogen activity	E	1

### Analysis of level 1 GO terms

**Fig 1:**
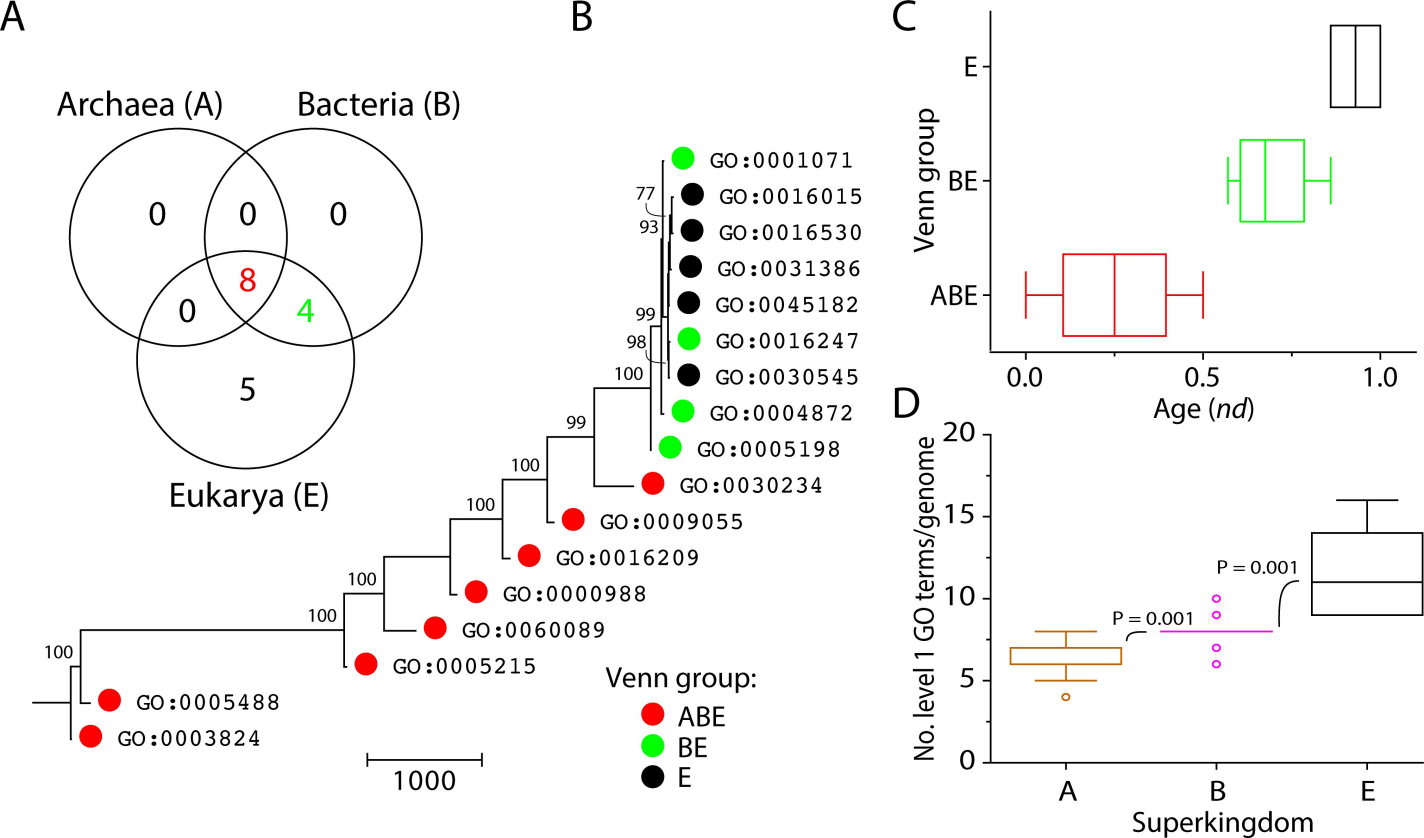
The distribution and evolution of level 1 GO terms in functionomes. (A) A Venn diagram illustrates the distribution patterns of level 1 molecular functions in the three superkingdoms, Archaea (A), Bacteria (B), and Eukarya (E). (B) The phylogenomic tree (tree length = 6820 steps) portraying the evolution of level 1 GO terms, with Venn distribution group-labeled leaves. Nonparametric bootstrap support (BS) values are shown above branches, which are supported by more than 50% of 1,000 replications. The tree was rooted by the Lundberg method. RI = retention index = 0.95, CI = consistency index = 0.85, *H*_*i*_ = homoplasy index = 0.15, and *g*_1_ = gamma distribution parameter = –0.72. (C) Boxplots shows the distribution of level 1 GO terms along the timeline in the three taxonomic groups. (D) Boxplots shows the number of level 1 GO terms per genome. Comparisons are significant at 0.001. A: Archaea, B: Bacteria, E: Eukarya.

A Venn diagram showed the distribution patterns of level 1 molecular functions based on the mapping of GO_TMF_ terms in the three superkingdoms (Fig 1). These included level 1 GO terms that were specific to Eukarya (E Venn taxonomic group), were shared by superkingdoms Bacteria and Eukarya (BE group), or were present in the three superkingdoms (ABE group).

Values of genomic abundance per taxon (*g*) ranged widely and had wide variances ranging 0–12,370 at level 1 and 0–10,150 at level 2 of the GO hierarchy of the DAG_mf_. The normalization of *g* values is explained in methods. We then produced normalized matrices for datasets of level 1 and level 2 GO terms for phylogenetic reconstruction of ToFs for both GO levels of the DAG_mf_. We then explored the origin of GO_TMF_ terms linked to higher level GO_MF_ terms by builfing a ToF of terminal GO terms, deriving a timeline of their ages using phylogenomic analysis, and mapping these ages to the higher level 1 and level 2 GO terms.

**Fig 2:**
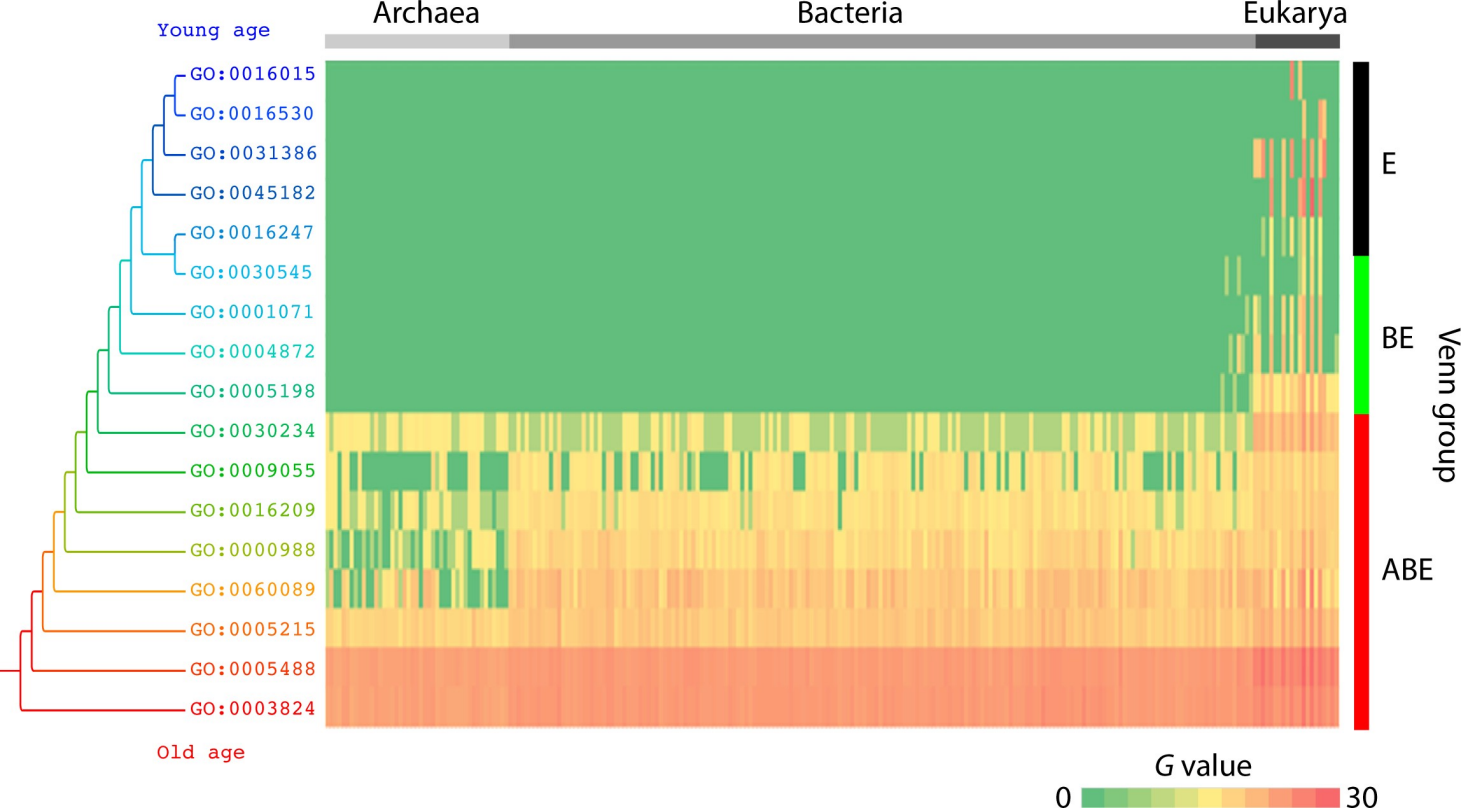
The heat map defines the distribution of genomic abundances (*g*) of 17 level 1 GO terms in the 249 organisms along the timeline (*nd*).

The eight basal taxa in the tree were shared by the three superkingdoms (ABE) and included GO:0003824–catalytic activity, GO:0005488–binding, GO:0005215–transporter activity, GO:0060089–molecular transducer activity, GO:0000988–protein binding transcription factor activity, GO:0016209–antioxidant activity, GO:0009055–electron carrier activity, and GO:0030234–enzyme regulator activity. All of these basal bifurcations were robust [99–100% nonparametric bootstrap support (BS)], indicating these level 1 GO terms are the most ancient molecular functions. On the other hand, GO:0005198–structural molecule activity, GO:0004872–receptor activity, GO:0001071–nucleic acid binding transcription factor activity, and GO:0016247–channel regulator activity were shared by Bacteria and Eukarya (BE). The most recent molecular functions were uniquely present in Eukarya (E) and included GO:0045182–translation regulator activity, GO:0030545–receptor regulator activity, GO:0031386–protein tag, GO:0016530–metallochaperone activity, and GO:0016015–morphogen activity. We also measured the age of individual molecular functions in a relative 0–1 scale defined by node distance (*nd*) from the hypothetical ancestor of molecular functions in the tree, which provides phylogeny-embedded information in timelines [17]. Remarkably, catalytic activity and binding level 1 GO terms appeared very early (*nd* < 0.1) (Table 2). Boxplots showed the appearence of Venn taxonomic groups based on level 1 of the DAG_mf_ (Fig 1C). We observed that the most ancient level 1 GO terms (0 ≤ *nd* ≤ 0.5) were universal and shared by the ABE Venn group and followed by the appearence of the BE and E groups, respectively. Relatively new level 1 GO terms were not uniquely present in Archaea (there is no A taxonomic group), suggesting that the BE and E groups increased novel functional activities during evolution, the likely result of bacterial biodiversity and eukaryal multicellularity. Data in Venn taxonomic group distributions is consistent with a scenario in which functions evolved from a complex and rich ancestor of extant life, producing archaeal functionomes first by massive genome streamlining [21]. Eukarya unique terms appeared in the very late phase of the timeline (0.86 ≤ *nd* ≤1). This also indicates that unique Eukarya level 1 functions are crucial, given the number of genomes that were sampled (21 eukaryal genomes). The number of level 1 GO terms range 4–8 per archaeal genome while ranging 6–10 per bacterial genome and 9–16 per eukaryal genome (Fig 1D). Thus, the number of high order molecular functions increased in the order Archaea, Bacteria and Eukarya. The color array of the evolutionary heat map describes the distribution of genomic abundances (*g*) of the 17 level 1 GO terms in the 249 organisms that were investigated (Fig 2). The result suggests early saturation of genomic abundance in the timeline (0 ≤ *nd* ≤ 0.5). The topography of the heat map indicated that the majority of genomic abundance belonged to catalytic activity and binding GO terms in the three superkingdoms. In contrast, high abundances of relatively new terms were also observed in Bacteria and Eukarya. Remarkably, BE-specific level 1 functions did not appear in all bacteria. Structural molecule activity appeared in *Chloroflexus aurantiacus* (Chloroflexi), *Lactobacillus delbrueckii* (Firmicutes), and *Maricaulis maris* (α-Proteobacteria), receptor activity appeared in *Opitutus terrae* (Verrucomicrobia), *Haliangium ochraceum* (δ-Proteobacteria), *Anaeromyxobacter dehalogenans* (δ-Proteobacteria), nucleic acid binding transcription factor activity appeared in *Opitutus terrae* (Verrucomicrobia) and *Herminiimonas arsenicoxydans* (β-Proteobacteria), and channel regulator activity appeared in *Salinispora arenicola* (Actinobacteria) and *Haliangium ochraceum* (δ-Proteobacteria). The high abundance and rarity of these BE functions in bacteria as an organismal group, suggests they likely resulted from loss by reductive evolution in microbes (including complete loss in Archaea) rather than gain in Bacteria and Eukarya, or less likely, by massive horizontal transfer between Eukarya and Bacteria.

**Fig 3:**
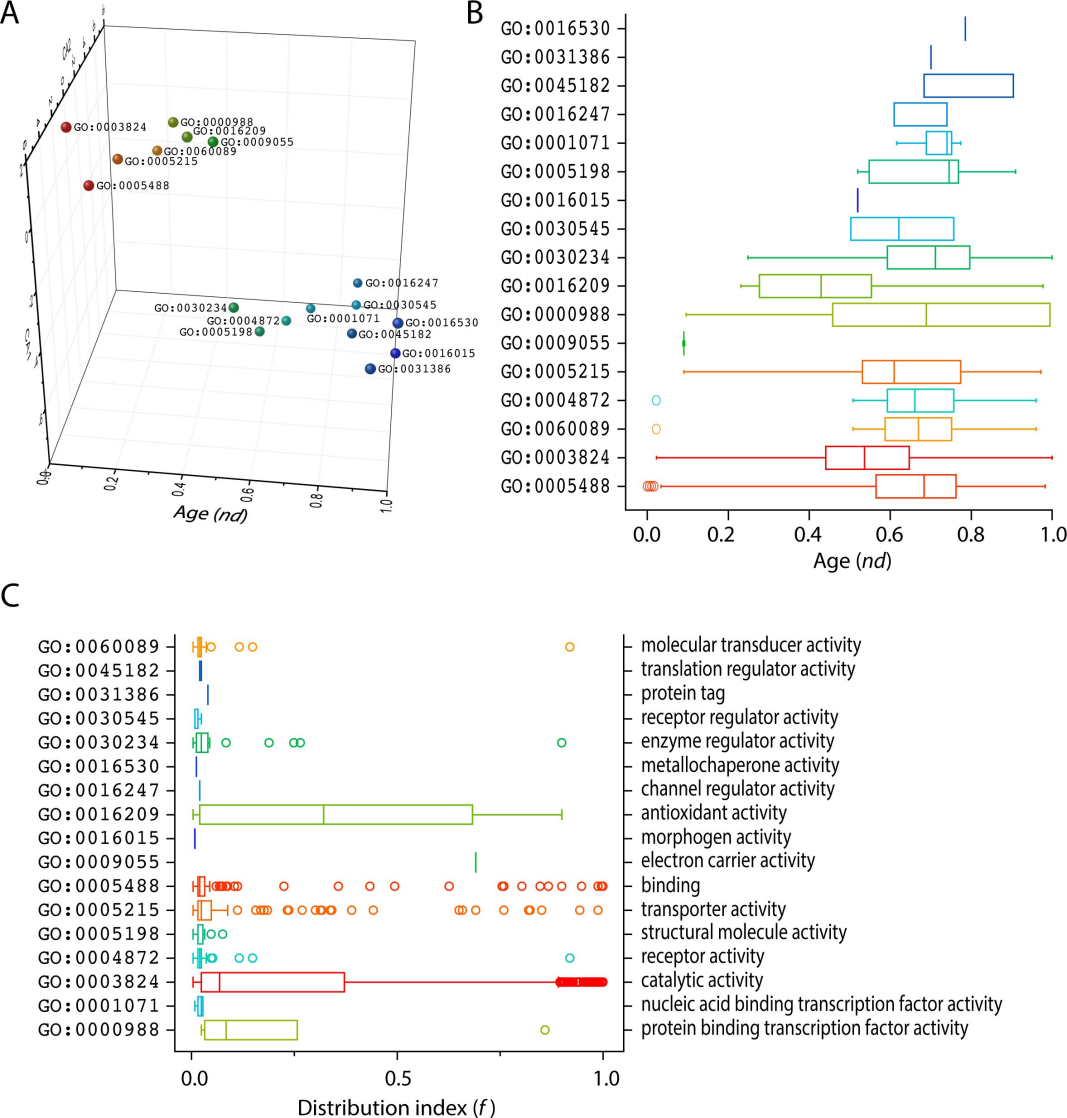
Evolution of level 1 molecular functions. (A) Correspondence Analysis (CA) of GO_MF_ explains clusters of level 1 GO terms with respect to age (*nd*) and CA values. (B) Boxplot displaying the distribution of level 1 GO terms with respect to age (*nd*) in GO_TMF_ terms of 249 organisms. (C) Boxplot displaying the distribution index of terminal terms categorized by level 1 GO terms.

To further explore the existence of evolutionary patterns of level 1 GO terms in the ToF along with the timelines reconstructed from the GO_TMF_ dataset, we performed both correspondence analysis (CA) and principal component analysis (PCA) of level 1 GO terms and represented molecular functions in 3D plots using evolutionary age (*nd* value) and the first two components of the multivariate statistical descriptive techniques as plot axes. Remarkably, two clear groups of level 1 terms were evident in the CA and PCA 3D representation, fundamentally defined by the age axis and in the case of the CA representation, the CA1-2 loading (Fig 3A). Note that both CA and PCA assume a multivariate normal or log-normal distribution, which is far from a universal feature of biological data. In particular, 7 out of the 8 universal terms shared by the ABE taxonomic group were clustered together. The only exception was GO:0030234–enzyme regulator activity. This finding suggests that genes encoding enzymes which are essential for catalysis are much more frequent than genes that encode various structural and regulatory proteins [45]. This may stem from processes of evolutionary divergence and convergence that can affect reduction and expansion of repertoires, together with processes of recruitment that would change the functional makeup of an organism [17]. To investigate the historical relationship between the two major groups of level 1 GO terms, we calculated the degree of monophyly (GSI) for the groups. GSI values close to 1 indicate a tendency towards monophyly of a given group whereas values close to zero indicate a tendency towards dispersal [8]. The group of 7 universal level 1 GO terms mentioned above had a GSI value of 0.77 while the other group had a value of 1, suggesting increased dispersal forces of the basal universal group probably stemming from increased diversification and recruitment. The boxplot showed the appearence order of GO_TMF_ assigned to level 1 of the DAG_mf_ in the evolutionary timeline (Fig 3B). The first molecular function at terminal level (GO_TMF_) was mapped to binding (GO:0005488) and was followed by catalytic activity (GO:0003824) in evolution. However, when we pursued a top-down phylogenetic approach to clarify the origin of molecular functions at the highest level, catalytic activity was the most ancient level 1 term of the DAG_mf_ (Fig 1B), and observation that was also compatible with corresponding levels of genomic abundance (Fig 2). Thus, we propose that level 1 GO terms diversified from a pool of ancient non-specific functions much earlier than organismal diversification, the appearance of distinct Venn taxonomical groups, and the massive rise of regulatory mechanisms in biology. This was already intimated by the mapping of lower order GO terms to higher classification, which showed that the diversification of ancient catalytic activity at lowest level of the DAG_mf_ was replaced by more specific and advanced functions such as ATP binding (GO:0005524) [21].

We compared the distribution indexes (*f*) of terminal GO_TMF_ terms mapping to level 1 GO terms of the DAG_mf_, which portray the distributions of functional terms in the functionomes that were sampled. Interestingly, in addition to the distributions of terminal terms of catalytic activity and binding, the *f* values of antioxidant activity and transporter activity terminal terms also appeared considerably biased, spanning from complete to almost complete coverage to presence in only selected organisms (Fig 3C). This confirms the ancient origin of these level 1 GO terms, which have been the subject of continued recruitment in evolution; variants of these functions described as modern terminal terms accumulated throughout the timeline. We thus assume that ancient molecular functions likely embedded several primordial features distilled by the more ancient level 1 GO definitions of catalytic activity and binding, antioxidant activity, and transporter activity.

**Fig 4:**
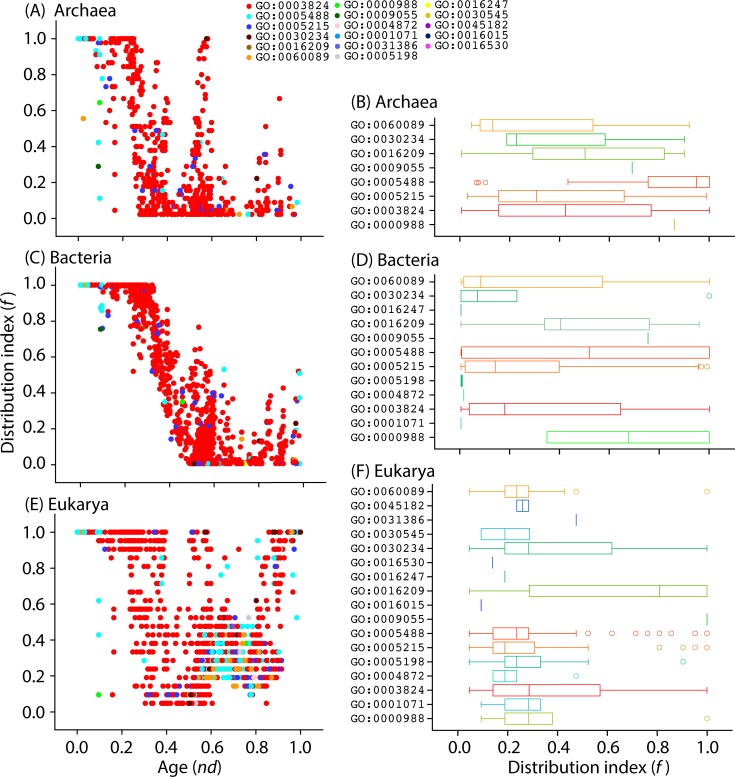
Order of the evolutionary appearance of level 1 GO terms in superkingdoms. (A) Scatter plot highlighting the distribution of level 1 GO terms in Archeae with respect to age (*nd*) and distribution in GO_TMF_ terms. (B) Boxplots displaying distribution index of terminal terms in each level 1 category in Archaea. (C) Scatter plot highlighting the distribution of level 1 GO terms in Bacteria with respect to nd and distribution in GO_TMF_ terms. (D) Boxplots displaying distribution index of terminal terms in each level 1 category in Bacteria. (E) Scatter plot highlighting the distribution of level 1 GO terms in Eukarya with respect to nd and distribution in GO_TMF_ terms. (F) Boxplots displaying distribution index of terminal terms in each level 1 category in Eukarya.

**Fig 5:**
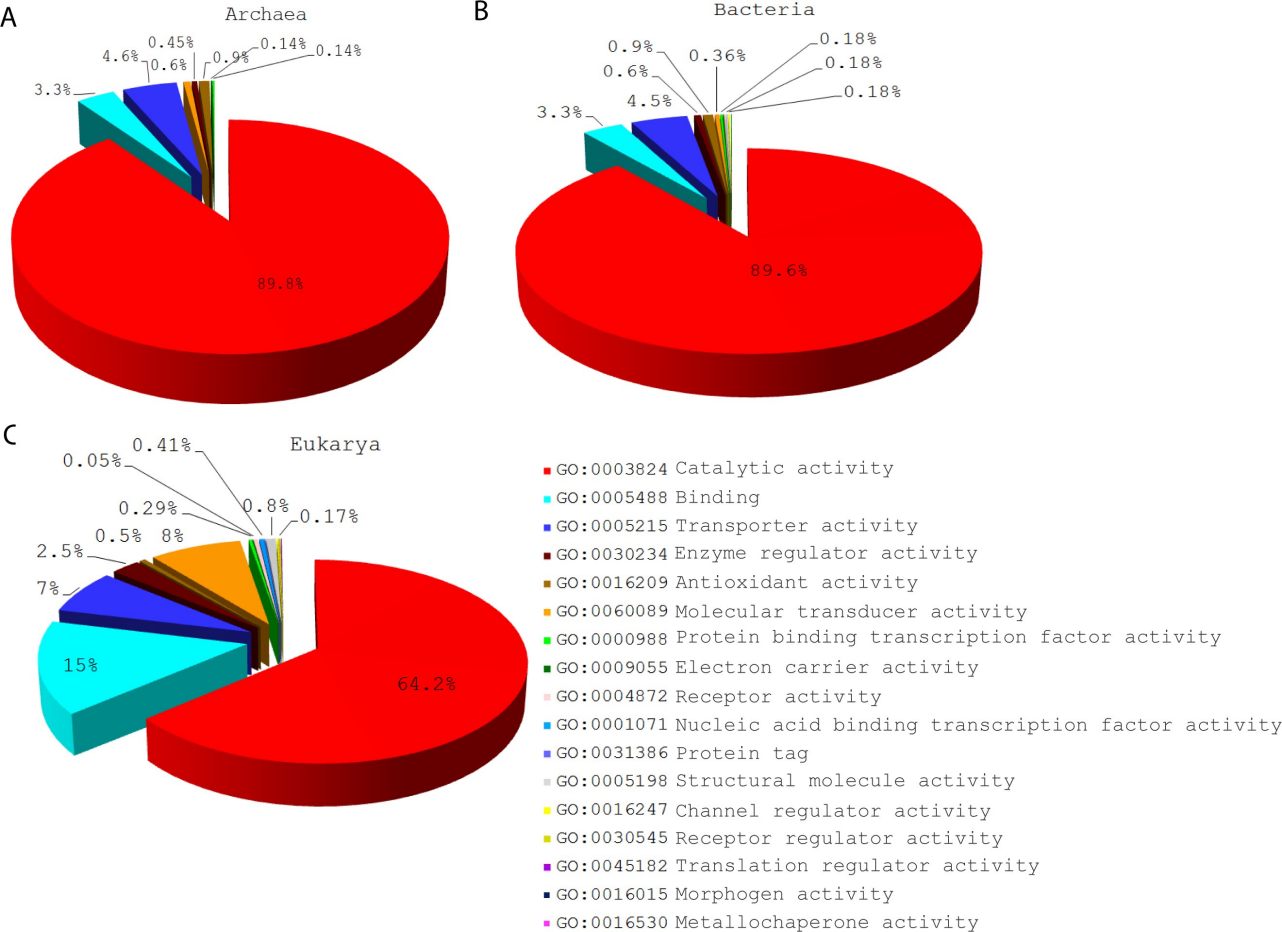
Distribution of terminal terms corresponding to level 1 GO terms of DAG_mf_ in the functionomes of the three superkingdoms of life. (A) Pie chart of Archaea terminal GO terms corresponded to level 1 of of DAG_mf_. (B) Pie chart of Bacteria terminal GO terms corresponded to level 1 of of DAG_mf_. (C) Pie chart of Eukarya terminal GO terms corresponded to level 1 of DAG_mf_.

The timeline of terminal GO terms also uncovered the order of appearance of level 1 molecular functions and their distribution in the functionomes of the different superkingdoms. We plotted the ages (*nd* values) of level 1 GO terms against the distribution index *f* in each taxonomic group (Fig 4). Recall that, operationally, *f* represents the total number of genomes encoding a GO_mf_ term divided by the total number of genomes. It explains popularity of GO terms among the organisms sampled from the three superkingdoms. The graph showed prominent evolutionary patterns. In Archaea, the majority of terminal GO terms mapped to level 1 of the DAG_mf_ corresponded to GO:0003824–catalytic activity (89.8%), followed by GO:0005215–transporter activity (4.6%) (Fig 5A). Binding (3.3%), catalytic activity, and GO:0030234–enzyme regulator activity had high *f* value equal to 1 early in the evolutionary timeline of terminal terms (0 ≤ *nd* ≤0.25) (Fig 4A). When comparing the archaeal distribution index in the molecular function dataset using boxplot representations of *f* value spread, binding function (GO:0005488) had a tendency (median) of over-representation approaching *f* = ~1 (Fig 4B). On the other hand, catalytic activity (GO:0003824) was widespread in archaeal proteomes (0 < *f* ≤ 1). Interestingly, protein binding transcription factor activity (GO:0000988) was also over-represented in Archaea. In addition, all of these functions were universally shared functions (ABE) that had in general higher median values than Bacteria and Eukarya, suggesting that Archaea followed a parsimonious strategy for functional toolkits while Bacteria and Eukarya gained a huge number of novel functions [21].

In the case of Bacteria, the distributions of GO_TMF_ terms assigned to level 1 GO terms of the DAG_mf_ were similar to those of Archaea (Fig 4C). The majority of terminal GO terms mapping to level 1 belonged to catalytic activity (89.6%) followed by transporter activity (4.5%) (Fig 5B) just as those of Archaea. Again, the representation of binding function (3.3%) was lower than transporter activity. Remarkably, the five universal functions (catalytic activity, binding, enzyme regulator activity, GO:0060089–molecular transducer activity, and GO:0000988–protein binding transcription factor activity) had high *f* value equal to 1 in the 0≤ *nd* ≤0.25 period of the timeline (Fig 4C). However, the *f* value started to drop with an increase in *nd* value by the end of that period. Catalytic activity, binding, transporter activity, and molecular transducer activity were widespread (0< *f* ≤1) in bacterial proteomes (Fig 4D). The relatively lower median *f* values in Bacteria may be explained by genome reduction events as well as the development of some bacterial specific traits.

Eukarya showed variability in the distribution of level 1 GO terms. All universal level 1 GO terms (shared by the Venn ABE taxonomic group) had *f* = 1 in the 0 ≤ *nd* ≤0.25 age range. However, protein binding transcription factor activity function appeared at *nd* = 0.99 (Fig 4E). Catalytic activity (64.2%) dominated the spread of GO_TMF_ terms (Fig 5C), followed by binding function (15%) instead of transporter activity, which was the second most common function in Archaea and Bacteria. Catalytic activity, antioxidant activity, and enzyme regulator activity had high representation (0< *f* ≤1) in eukaryotes (Fig 4F). Interestingly, the representation of binding functions decreased while electron carrier activity (GO:0009055) was acquired by all eukaryotes (*f* = 1). Overall, these patterns of age and organismal distribution suggest that the last universal common ancestor (urancestor) of superkingdoms had a cell-like amitochondriate and functionally complex structure with complex catalytic machinery already present, as proposed by previous studies of protein domains and molecular functions [17, 30, 42, 46]. They also suggest that eukaryotes gained novel molecular functions during evolution, explaining the huge molecular and organismal diversity at all levels of organization present in Eukarya [21].

### Analysis of level 2 GO terms

**Fig 6:**
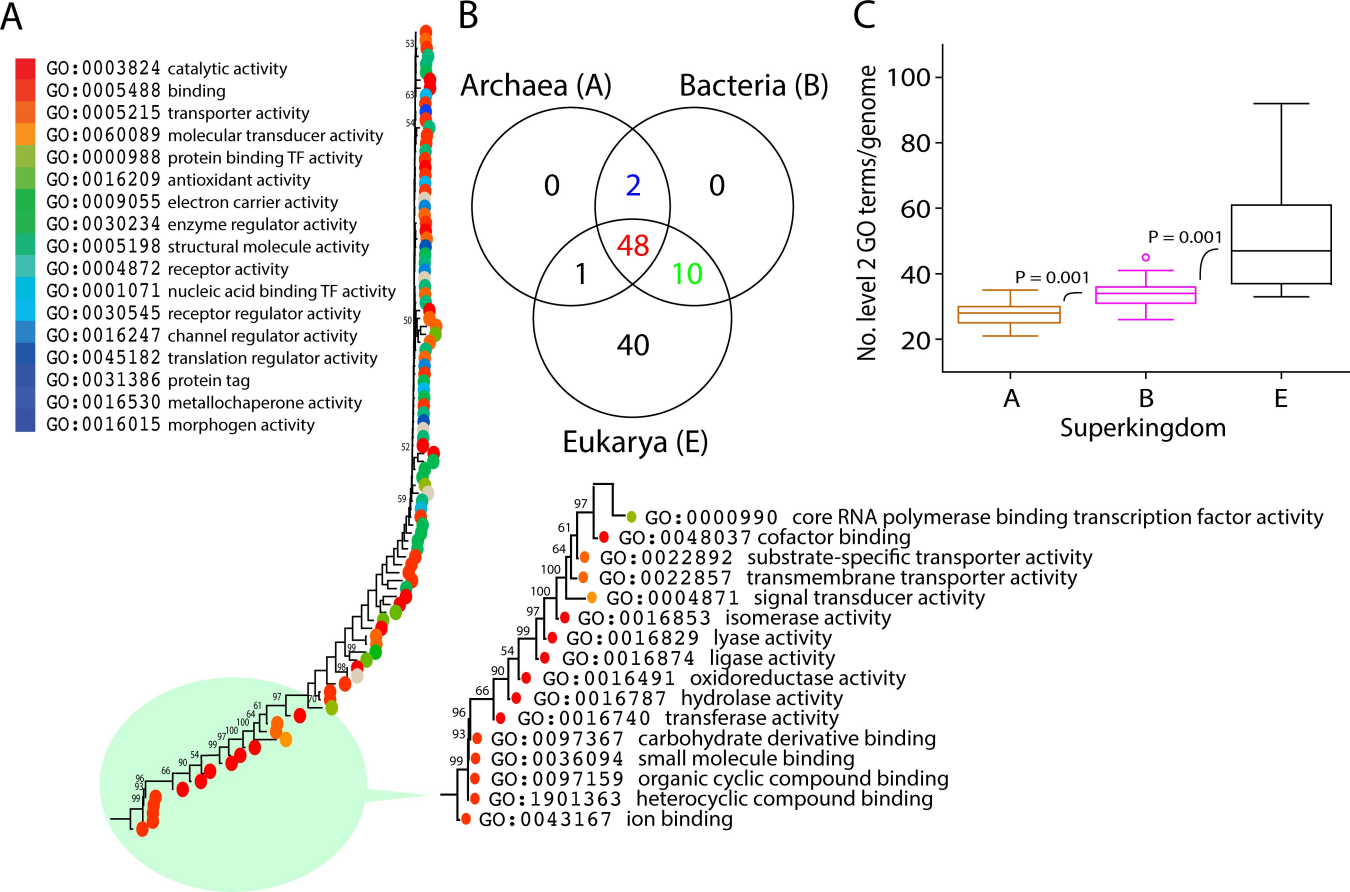
Evolution of level 2 GO terms. (A) A ToF potraying evolution of level 2 GO terms (Tree length = 12,363). Molecular functions are colored according to corresponding level 1 GO terms. Nonparametric BS values are shown above branches, which are supported by more than 50% of 1,000 replications. The tree was rooted by the Lundberg method. Tree length = 12,363, RI = 0.92, CI = 0.46, Hi = 0.53, and g1 = -0.67. (B) Venn diagram illustrates the distribution numbers of level 2 molecular functions in the three superkingdoms. (C) Boxplots shows the number of level 2 GO terms per genome. Comparisons are significant at 0.001. A: Archaea, B: Bacteria, E: Eukarya.

A total of 101 out of 174 molecular functions exist at level 2 of the DAG_mf_ according to the taxon selection criteria (Table 3). In this set, 2 of 17 level 1 molecular functions had no level 2 associated taxa, including GO:0031386–protein tag and GO:0016015–morphogen activity. A Venn diagram showed the distribution patterns of level 2 molecular functions based on correspondence of GO_TMF_ terms in the three superkingdoms (Fig 6B). These included level 2 GO terms that were exclusive to Eukarya (40), were shared by the AB (2), AE (1), and BE (10) Venn taxonomic groups or were present in three the superkingdoms (ABE; 48). Regarding the number of level 2 GO terms per genome, it ranged 21–35 per archaeal genome and 26–45 per bacterial genome (Fig 6C). Again, this number is larger in Eukarya, ranging 33–92 per eukarial genome. These ranges again increased in the order Archaea, Bacteria and Eukarya.

**Table 3:** List of level 2 GO terms mapped to GO_TMF_ terms, sorted by *nd* values.

GO accession (Level 2)	Definition	Venn group	*nd*
GO:0097159	organic cyclic compound binding	ABE	0.02
GO:1901363	heterocyclic compound binding	ABE	0.02
GO:0036094	small molecule binding	ABE	0.03
GO:0097367	carbohydrate derivative binding	ABE	0.05
GO:0016740	transferase activity	ABE	0.07
GO:0016787	hydrolase activity	ABE	0.08
GO:0016491	oxidoreductase activity	ABE	0.1
GO:0016874	ligase activity	ABE	0.12
GO:0016829	lyase activity	ABE	0.14
GO:0016853	isomerase activity	ABE	0.15
GO:0004871	signal transducer activity	ABE	0.17
GO:0022857	transmembrane transporter activity	ABE	0.19
GO:0022892	substrate-specific transporter activity	ABE	0.2
GO:0048037	cofactor binding	ABE	0.22
GO:0000990	core RNA polymerase binding transcription factor activity	ABE	0.24
GO:0008144	drug binding	ABE	0.27
GO:0033218	amide binding	ABE	0.27
GO:1901681	sulfur compound binding	ABE	0.27
GO:0019239	deaminase activity	ABE	0.31
GO:0038023	signaling receptor activity	ABE	0.31
GO:0004601	peroxidase activity	ABE	0.32
GO:0004129	cytochrome-c oxidase activity	ABE	0.32
GO:0019534	toxin transporter activity	ABE	0.34
GO:0070283	radical SAM enzyme activity	ABE	0.34
GO:0090484	drug transporter activity	ABE	0.34
GO:0004784	superoxide dismutase activity	ABE	0.36
GO:0004791	thioredoxin-disulfide reductase activity	ABE	0.39
GO:0004133	glycogen debranching enzyme activity	ABE	0.41
GO:0005515	protein binding	ABE	0.41
GO:0008430	selenium binding	ABE	0.41
GO:0008987	quinolinate synthetase A activity	ABE	0.41
GO:0060589	nucleoside-triphosphatase regulator activity	ABE	0.41
GO:0030246	carbohydrate binding	ABE	0.42
GO:0008289	lipid binding	ABE	0.44
GO:0019207	kinase regulator activity	ABE	0.46
GO:0008047	enzyme activator activity	E	0.47
GO:0004857	enzyme inhibitor activity	ABE	0.49
GO:0061134	peptidase regulator activity	ABE	0.51
GO:0060090	binding, bridging	E	0.53
GO:0003700	sequence-specific DNA binding transcription factor activity	BE	0.54
GO:0005212	structural constituent of eye lens	E	0.56
GO:0000989	transcription factor binding transcription factor activity	ABE	0.59
GO:0010576	metalloenzyme regulator activity	BE	0.59
GO:0038024	cargo receptor activity	ABE	0.59
GO:0008265	Mo-molybdopterin cofactor sulfurase activity	E	0.63
GO:0019208	phosphatase regulator activity	ABE	0.63
GO:0008307	structural constituent of muscle	E	0.64
GO:0009975	cyclase activity	ABE	0.64
GO:0030337	DNA polymerase processivity factor activity	AE	0.64
GO:0038187	pattern recognition receptor activity	E	0.66
GO:0090079	translation regulator activity, nucleic acid binding	E	0.68
GO:0019911	structural constituent of myelin sheath	E	0.69
GO:0050997	quaternary ammonium group binding	E	0.71
GO:0010851	cyclase regulator activity	E	0.73
GO:0030235	nitric-oxide synthase regulator activity	E	0.75
GO:0030546	receptor activator activity	E	0.75
GO:0015643	toxic substance binding	BE	0.76
GO:0016248	channel inhibitor activity	BE	0.78
GO:0001618	virus receptor activity	E	0.81
GO:0051183	vitamin transporter activity	E	0.81
GO:0005201	extracellular matrix structural constituent	E	0.83
GO:0005487	nucleocytoplasmic transporter activity	E	0.85
GO:0017080	sodium channel regulator activity	BE	0.85
GO:0005055	laminin receptor activity	E	0.86
GO:0005246	calcium channel regulator activity	BE	0.86
GO:0005326	neurotransmitter transporter activity	E	0.86
GO:0017056	structural constituent of nuclear pore	E	0.86
GO:0097493	structural molecule activity conferring elasticity	E	0.86
GO:0032947	protein complex scaffold	E	0.88
GO:0043028	cysteine-type endopeptidase regulator activity involved in apoptotic process	E	0.88
GO:0005549	odorant binding	E	0.9
GO:0019825	oxygen binding	E	0.9
GO:0030548	acetylcholine receptor regulator activity	E	0.9
GO:0032451	demethylase activity	ABE	0.9
GO:0042165	neurotransmitter binding	E	0.9
GO:0000386	second spliceosomal transesterification activity	E	0.92
GO:0003682	chromatin binding	E	0.92
GO:0030371	translation repressor activity	E	0.92
GO:0051184	cofactor transporter activity	ABE	0.92
GO:0008641	small protein activating enzyme activity	E	0.93
GO:0035804	structural constituent of egg coat	E	0.93
GO:0042910	xenobiotic transporter activity	ABE	0.93
GO:1901505	carbohydrate derivative transporter activity	ABE	0.93
GO:0003823	antigen binding	E	0.95
GO:0016531	copper chaperone activity	E	0.95
GO:0019808	polyamine binding	E	0.95
GO:0042562	hormone binding	E	0.95
GO:1901476	carbohydrate transporter activity	ABE	0.95
GO:0004362	glutathione-disulfide reductase activity	ABE	0.95
GO:0005200	structural constituent of cytoskeleton	BE	0.97
GO:0043021	ribonucleoprotein complex binding	BE	0.97
GO:0000035	acyl binding	E	0.98
GO:0015607	fatty-acyl-CoA transporter activity	E	0.98
GO:0030547	receptor inhibitor activity	E	0.98
GO:0001871	pattern binding	BE	1
GO:0005199	structural constituent of cell wall	BE	1
GO:0005213	structural constituent of chorion	E	1
GO:0008907	integrase activity	AB	1
GO:0009009	site-specific recombinase activity	AB	1
GO:0042979	ornithine decarboxylase regulator activity	E	1

**Fig 7:**
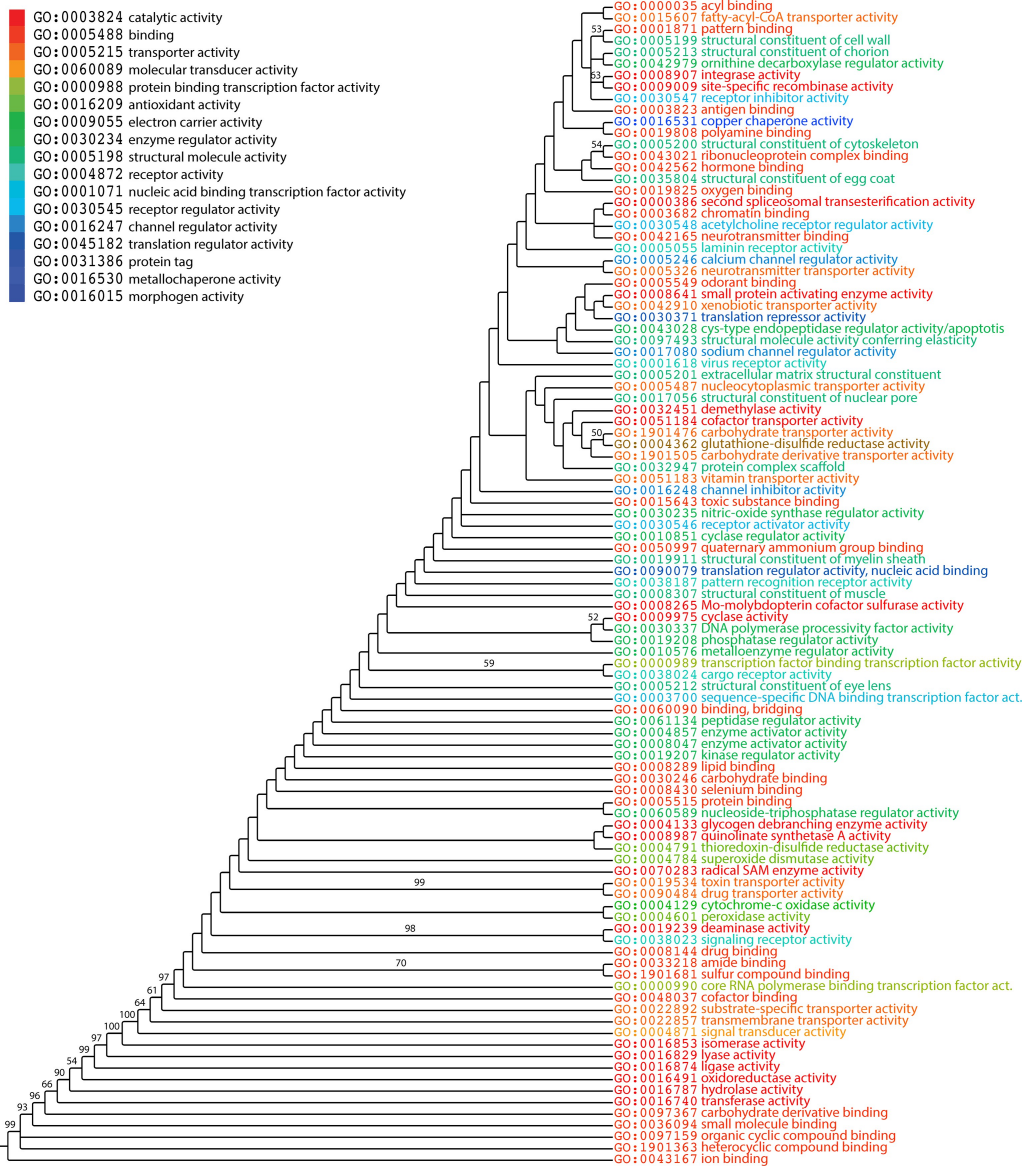
A ToF potraying the evolution of level 2 GO terms. Molecular functions are colored according to corresponding level 1 GO terms. Nonparametric BS values are shown above branches, which are supported by more than 50% of 1,000 replications. The tree was rooted by the Lundberg method. Tree length = 12,363; RI = 0.92; CI = 0.46, Hi = 0.53; and g1 = -0.67.

**Fig 8:**
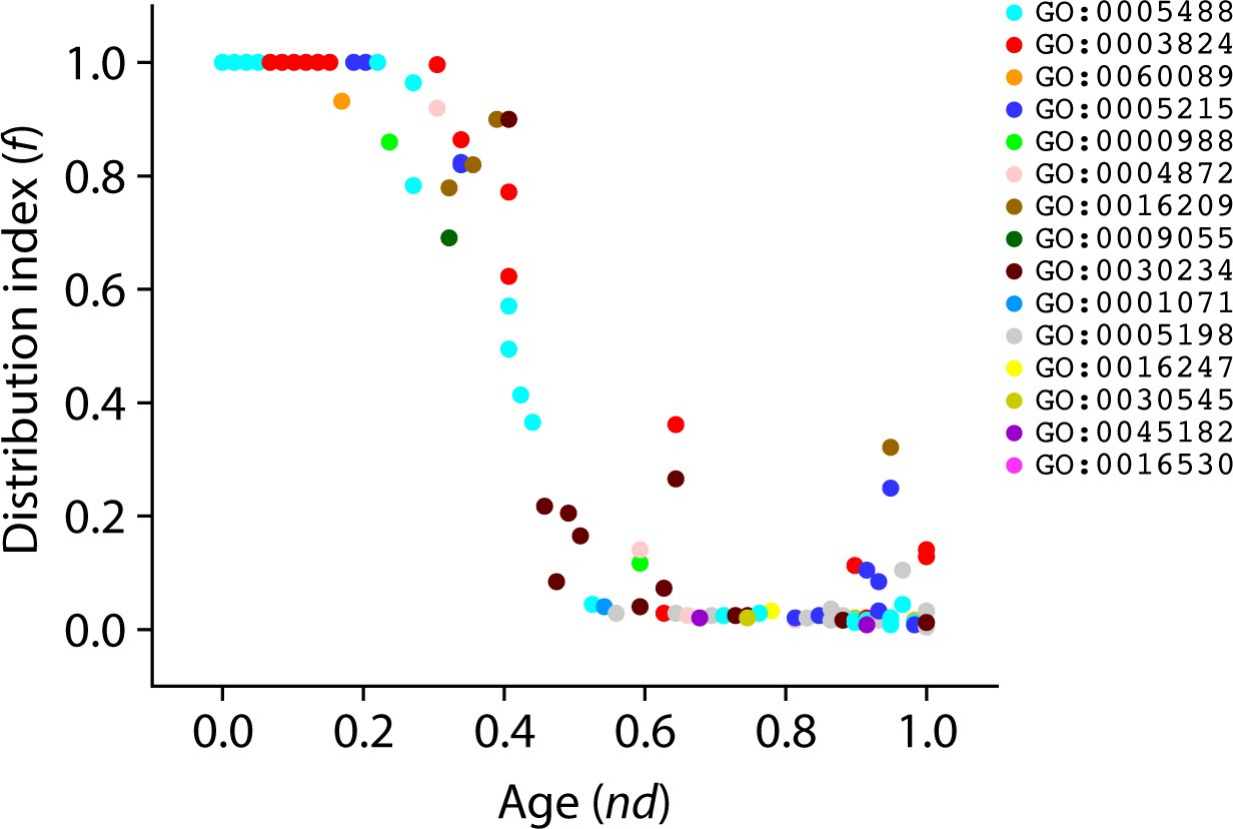
Order of the evolutionary appearence of level 2 GO terms. Scatter plot highlighting the distribution of level 2 GO terms with respect to age (*nd*) and molecular functions are colored according to corresponding level 1 GO terms.

**Fig 9:**
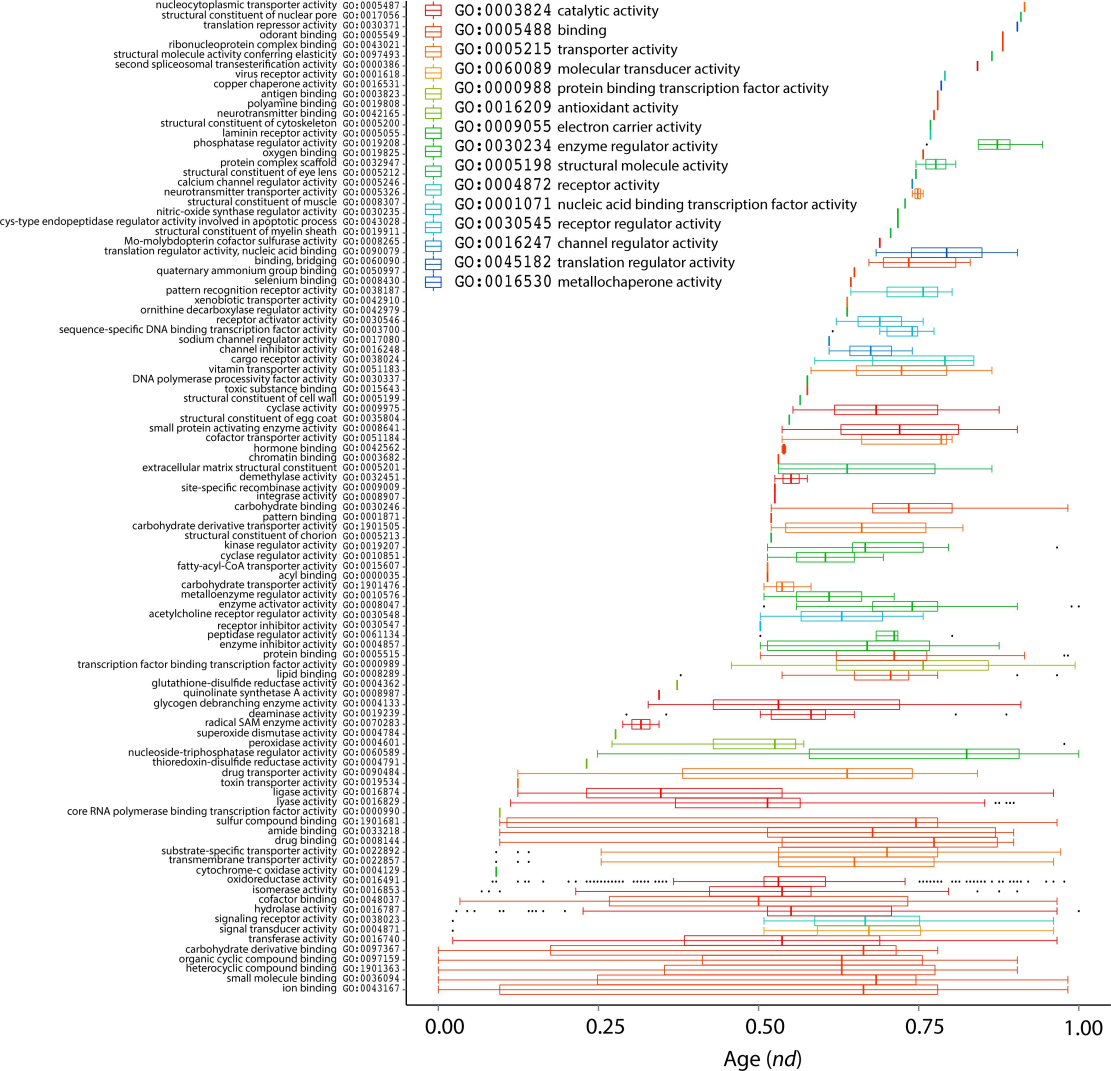
Boxplot displaying the distribution of level 2 GO terms with respect to age (*nd*) in GO_TMF_ terms of 249 organisms.

Phylogenomic analysis of level 2 GO terms generated a single most parsimonious ToF (Fig 6A). In general, higher retention index (RI) values represent better fit of phylogenetic characters to the phylogeny and thus lower probability of of non-vertical inheritance [8]. As expected in trees of this size, internal nodes are poorly supported by BS values. However, basal nodes were highly robust (BS>50%), especially close to the base of the tree. The most basal level 2 GO terms corresponded to binding, catalytic activitiy, and molecular transducer activity, in that order (Fig 7). Again, the first molecular functions at level 2 GO terms were mapped to binding, including ion binding (GO:0043167), heterocyclic compound binding (GO:1901363), organic cyclic compound binding (GO:0097159), small molecule binding (GO:0036094), and carbohydrate derivative binding (GO:0097367) and were followed by catalytic activity (GO:0003824) in evolution just like those of the ToF describing evolution of terminal GO terms. This result contrasts with a previous study of 38 genomes, in which the most ancient terms belong to catalytic activity [17]. Similar to that study, the six most ancient level 2 GO terms mapping to catalytic activity covered all six enzymatic activities according to Enzyme Commission classification: oxidoreductase activity (GO:0016491), transferase activity (GO:0016740), hydrolase activity (GO:0016787), lyase activity (GO:0016829), isomerase activity (GO:0016853), and ligase activity (GO:0016874). The most ancient level 2 GO terms, in addition to those level 2 terms mentioned above, GO:0022857 (transmembrane transporter activity), GO:0022892 (substrate-specific transporter activity), GO:0048037 (cofactor binding) corresponding to binding, catalytic activity, and transporter activity were present in all proteomes (*f* = 1) and appeared in the 0 ≤ *nd*≤ 0.25 range (Fig 8). Also, GO:0001871–pattern binding, GO:0005199–structural constituent of cell wall, GO:0005213–structural constituent of chorion, GO:0008907–integrase activity, GO:0009009–site-specific recombinase activity, and GO:0042979–ornithine decarboxylase regulator activity were most derived. A decreasing trend was obvious for *f* with increasing *nd*. Moreover, an accumulation of functions was observed at *nd* ≥ 0.5 with *f* approaching 0. These results suggest a major episode of functional diversification occurring at *nd* ~ 0.5 in which members of ancestral functions diversified their functional repertoire. In fact, functional diversification starts when newer functions become differentially excluded in some species, resulting in substantial decreases of representation (*f* <1). This is one expected outcome of lineage diversification. The boxplot showed the progressive appearence of GO_TMF_ assigned to level 2 of the DAG_mf_ (Fig 9). The first five molecular functions at terminal level (GO_TMF_) were mapped to binding (GO:0005488) and were followed by catalytic activity (GO:0003824) in evolution.

**Fig 10:**
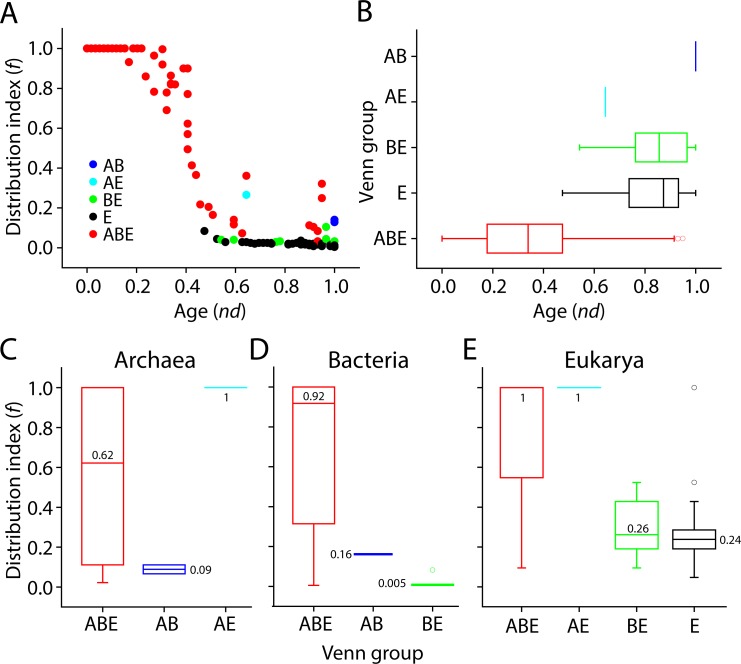
Evolution and distribution of level 2 GO terms. (A) Boxplots exhibit the distribution of level 2 GO terms with respect to evolutionary time and distribution in taxons. (B) Distribution index (f) uncovers evolutionary pattern of level 2 GO terms in taxonomic groups with respect to age (*nd*). (C) The distribution of level 2 molecular functions in Archaea in the Venn taxonomic groups. (D) The distribution of level 2 molecular functions in Bacteria in the Venn taxonomic groups. (E) The distribution of level 2 molecular functions in Eukarya in the Venn taxonomic groups.

The distribution of level 2 molecular functions in taxa along with age (*nd*) revealed that the functions of the Venn ABE group ranged from *nd* = 0 to *nd* ~ 1 and were followed by the appearance of the E, BE, AE, and AB groups, in that order (Fig 10A). The first molecular activity unique to Eukarya was GO:0008047–enzyme activator activity. The first molecular function shared by the BE group was GO:0003700–transcription factor activity, sequence-specific DNA binding. Remarkably, the only molecular function unique to the AE group was GO:0030337–DNA polymerase processivity factor activity. Processivity is defined as the ability of DNA polymerase to carry out continuous DNA synthesis on a template DNA while remaining topologically bound to it (from AmiGO). This suggests processive DNA templating began quite late in evolution, when Archaea and Eukarya began to diversify. A common processivity factor, clamp protein or clamp, exists in Bacteria, Archaea, and Eukarya [47]. Remarkably, archaeal and eukaryal clamps resemble each other structurally and in the way they perform their functions, which is highly similar in both superkingdoms [48–50]. The first molecular functions shared by the AB group were GO:0008907–integrase activity and GO:0009009–site-specific recombinase activity. Interestingly, Eukarya-specific molecular functions at level 2 appeared earlier than other Venn superkingdom-specific group taxa suggesting the primordial stem line of descent and/or diversification of Eukarya involved a eukaryal-like functional makeup. This exercise dissected functional chronologies for the three superkingdoms and revealed the important trend of a superkingdom loosing first a significant number of functions before engaging in functional innovation. Eukaryotes, in particular, developed a massive number of GO terms defined at level 2 of the DAG_mf_, thereby, compensating for early loss events. On the other hand, the ABE functional reportoire distributed with the highest median *f* values in the three superkingdoms (Fig 10C, 10D and 10E) and medians increased in the order Archaea (median *f* = 0.62), Bacteria (median *f* = 0.92), and Eukarya (median *f* = 1), with those of Archaea being considerably lower than Bacteria and Eukarya. The largest median values in all superkingdoms support the fundamental evolutionary assumption of the common ancestry of life [7]. The AB functions were poorly spread in Archaea and Bacteria (median *f* values 0.09 and 0.16). However, the AE function (GO:0030337) was highly represented in eukaryal and archaeal proteomes (median *f* value = 1) suggesting the existence of a strong horizontal trace of DNA polymerase processivity factor activity between Eukarya and Archaea. The BE functions were also poorly distributed in Bacteria (median *f* value = 0.005) while they were relatively higher in Eukarya (median *f* value = 0.26) indicating horizontal effects or biases in GO annotation. Finally, Eukarya-specific functions had slightly lower median *f* value (0.24) than those of the Venn BE taxonomic group in eukaryotes. These results provide a trace of ancient signal unifying Bacteria and Eukarya as sister groups in the ToL [7, 21].

The distribution index *f* of level 2 GO terms uncovered patterns embedded in the phylogenetic tree of level 2 GO terms. Fig 10 shows *f* values plotted against the relative age of functions (*nd*) labeled according to Venn taxonomic groups. The ancient level 2 GO terms were again present in all organisms examined (*f* = 1) and as expected their representation decreased along with increasing age. Eukarya-specific GO terms clustered at *nd* > 0.45 as their *f* approached 0. Interestingly, AB-specific GO terms were present at *nd* approaching 1. Conversely, BE-specific functions had very low *f* value and appeared at *nd* > 0.5 and there was only one function shared by the AE group. These results reinforce the notion that the tracing of the origins of the three superkingdoms from molecular functions based on hierarchical levels of GO terms and other considerations imply that the ancestor was eukaryotic-like and complex [9, 10, 30, 51, 52] contrary to the suggestion of Woese et al. [3]. Loss of molecular functions usually express as a decrease in usage (*f* value) of particular GO terms compared to older functions. Furthermore, and similar to molecular structures, the probability of loosing an existing function in a lineage later in evolution is more likely than the probability of other lineages independently discovering the same function at the time of its origin [30]. Therefore, trends of loss in the late appearences of BE, AE, and AB shared functions in addition to E specific functions suggest the emergence of lineages from the pool of the communal ancestor (Fig 10B).

### Analysis of level 3 GO terms

**Fig 11:**
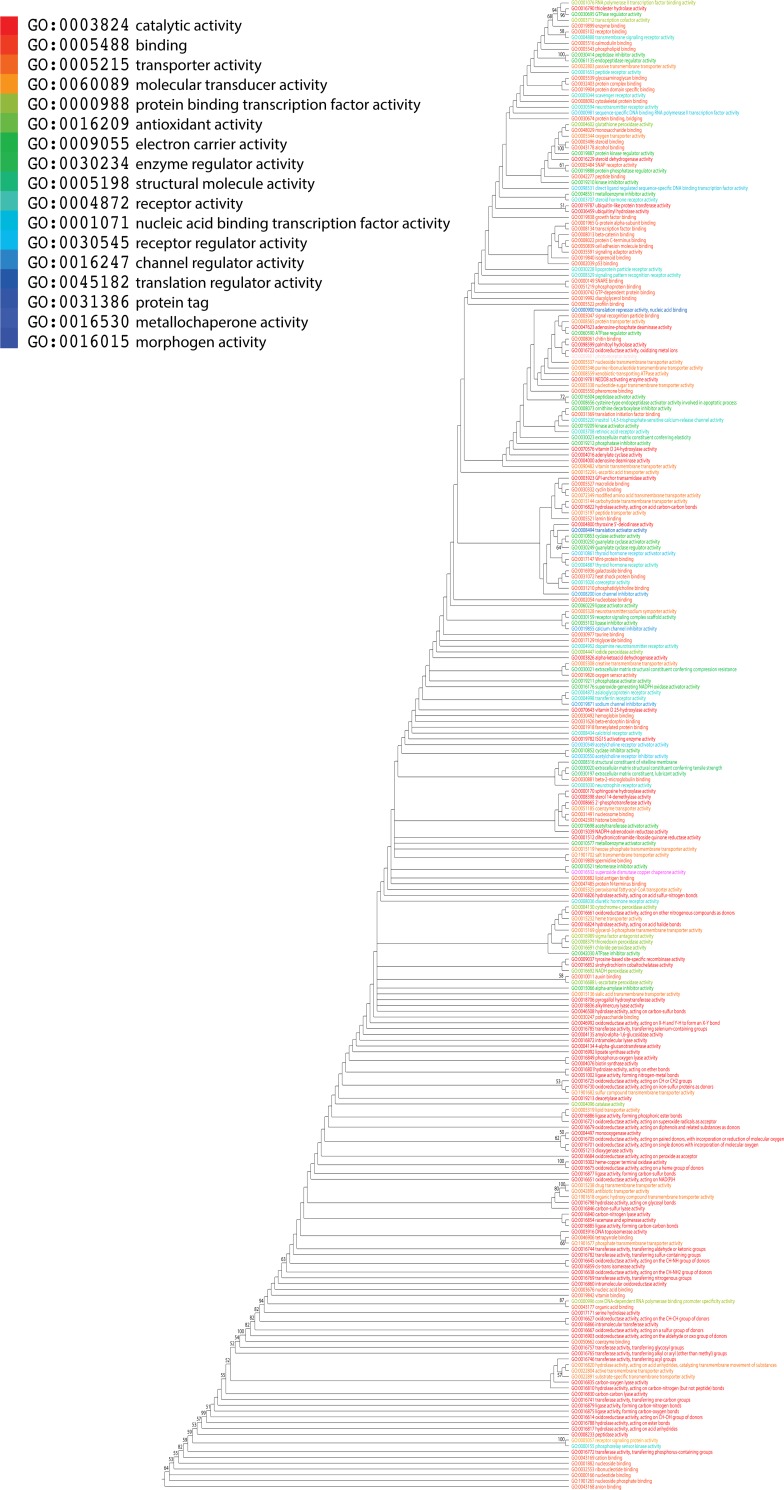
A ToF potraying evolution of level 3 GO terms. Molecular functions are colored according to corresponding level 1 GO terms. Nonparametric BS values are shown above branches, which are supported by more than 50% of 1,000 replications. The tree was rooted by the Lundberg method. Tree length = 24,364; RI = 0.88; CI = 0.24; *H*_*i*_ = 0.76; and *g*_1_ = -0.32.

We extracted level 3 GO terms annotated to terminal GO terms of the DAG_mf_. We found that 257 out of 742 level 3 GO terms correspond to 1891 terminal GO terms. As observed with level 2 GO terms, *g* values ranged 0–6,349 and were sufficient to extract evolutionary relationships and reconstruct a ToF at this level of the GO hierarchy. As expected in trees of this size, basal branches were moderately supported (generally BS < 50%) in the phylogenetic tree (Fig 11). We note that any children of “electron carrier activity” (GO:0009055) has no corresponding level 3 taxa in the AmiGO database. Therefore, GO:0004129–cytochrome-c oxidase activity has no associated level 3 GO terms. We also excluded “signaling receptor activity” (GO:0038023). Although it is a level 3 child of molecular transducer activity (GO:0060089), it is also a level 2 child of receptor activity (GO:0004872). Therefore, this molecular function was taken as child of receptor activity according to our taxa selection criteria. In the ToF, the most ancient molecular functions included anion binding, nucleoside phosphate binding, nucleotide binding, ribonucleotide binding, nucleoside binding, and cation binding (Table 4). All of these activities were related to binding function at level 1 of the DAG_mf_. Transferase activity, transferring phosphorus-containing groups, peptidase activity, and receptor signaling protein activity were the most ancient level 3 terms that followed. The youngest level 3 functions were GO:0030695 (GTPase regulator activity), GO:0004602 (glutathione peroxidase activity), GO:0016790 (thiolester hydrolase activity) and GO:0048029 (monosaccharide binding). Consistent with the ToF of level 2 GO terms, basal taxa were related to “binding” function followed by “catalytic activity”, “receptor activity” and, “molecular transducer activity” (Fig 11). Boxplots also showed the progressive appearence of GO_TMF_ assigned to level 3 of the DAGmf (S1 Fig). Similar to level 2 terms, the first six molecular functions at level 3 terms were mapped to binding function and were followed by catalytic activity in evolution.

**Table 4:** List of level 3 GO terms mapped to GO_TMF_ terms, sorted by *nd* values.

GO accession (Level 3)		Definition	Venn Taxon	nd
GO:0043168	anion binding		ABE	0
GO:1901265	nucleoside phosphate binding		ABE	0.0096
GO:0000166	nucleotide binding		ABE	0.0096
GO:0032553	ribonucleotide binding		ABE	0.0192
GO:0001882	nucleoside binding		ABE	0.0288
GO:0043169	cation binding		ABE	0.0385
GO:0016772	transferase activity, transferring phosphorus-containing groups		ABE	0.0481
GO:0008233	peptidase activity		ABE	0.0673
GO:0005057	receptor signaling protein activity		ABE	0.0769
GO:0016817	hydrolase activity, acting on acid anhydrides		ABE	0.0769
GO:0000155	phosphorelay sensor kinase activity		ABE	0.0769
GO:0016788	hydrolase activity, acting on ester bonds		ABE	0.0865
GO:0016614	oxidoreductase activity, acting on CH-OH group of donors		ABE	0.0962
GO:0016875	ligase activity, forming carbon-oxygen bonds		ABE	0.1058
GO:0016879	ligase activity, forming carbon-nitrogen bonds		ABE	0.1154
GO:0016741	transferase activity, transferring one-carbon groups		ABE	0.125
GO:0016830	carbon-carbon lyase activity		ABE	0.1346
GO:0016810	hydrolase activity, acting on carbon-nitrogen (but not peptide) bonds		ABE	0.1538
GO:0016765	transferase activity, transferring alkyl or aryl (other than methyl) groups		ABE	0.1538
GO:0016757	transferase activity, transferring glycosyl groups		ABE	0.1635
GO:0016835	carbon-oxygen lyase activity		ABE	0.1635
GO:0050662	coenzyme binding		ABE	0.1731
GO:0016746	transferase activity, transferring acyl groups		ABE	0.1731
GO:0022891	substrate-specific transmembrane transporter activity		ABE	0.1827
GO:0016903	oxidoreductase activity, acting on the aldehyde or oxo group of donors		ABE	0.1827
GO:0016667	oxidoreductase activity, acting on a sulfur group of donors		ABE	0.1923
GO:0022804	active transmembrane transporter activity		ABE	0.1923
GO:0016820	hydrolase activity, acting on acid anhydrides, catalyzing transmembrane movement of substances		ABE	0.1923
GO:0017171	serine hydrolase activity		ABE	0.2115
GO:0016627	oxidoreductase activity, acting on the CH-CH group of donors		ABE	0.2115
GO:0016866	intramolecular transferase activity		ABE	0.2115
GO:0019842	vitamin binding		ABE	0.2308
GO:0000996	core DNA-dependent RNA polymerase binding promoter specificity activity		ABE	0.2308
GO:0043177	organic acid binding		ABE	0.2308
GO:0003676	nucleic acid binding		ABE	0.2404
GO:0016860	intramolecular oxidoreductase activity		ABE	0.25
GO:0016769	transferase activity, transferring nitrogenous groups		ABE	0.2596
GO:0016638	oxidoreductase activity, acting on the CH-NH2 group of donors		ABE	0.2692
GO:0016859	cis-trans isomerase activity		ABE	0.2885
GO:0016645	oxidoreductase activity, acting on the CH-NH group of donors		ABE	0.2885
GO:0016782	transferase activity, transferring sulfur-containing groups		ABE	0.2885
GO:0016744	transferase activity, transferring aldehyde or ketonic groups		ABE	0.2981
GO:0003916	DNA topoisomerase activity		ABE	0.3173
GO:0016840	carbon-nitrogen lyase activity		ABE	0.3269
GO:0046906	tetrapyrrole binding		ABE	0.3269
GO:1901677	phosphate transmembrane transporter activity		ABE	0.3269
GO:0016651	oxidoreductase activity, acting on NAD(P)H		ABE	0.3365
GO:0016854	racemase and epimerase activity		ABE	0.3365
GO:0016885	ligase activity, forming carbon-carbon bonds		ABE	0.3365
GO:0016846	carbon-sulfur lyase activity		ABE	0.3365
GO:0016877	ligase activity, forming carbon-sulfur bonds		ABE	0.3462
GO:0016798	hydrolase activity, acting on glycosyl bonds		ABE	0.3462
GO:1901618	organic hydroxy compound transmembrane transporter activity		ABE	0.3558
GO:0016675	oxidoreductase activity, acting on a heme group of donors		ABE	0.3654
GO:0042895	antibiotic transporter activity		ABE	0.3654
GO:0016684	oxidoreductase activity, acting on peroxide as acceptor		ABE	0.3654
GO:0015238	drug transmembrane transporter activity		ABE	0.3654
GO:0015002	heme-copper terminal oxidase activity		ABE	0.3654
GO:0016679	oxidoreductase activity, acting on diphenols and related substances as donors		ABE	0.3846
GO:0051213	dioxygenase activity		ABE	0.3942
GO:0004497	monooxygenase activity		ABE	0.3942
GO:0016705	oxidoreductase activity, acting on paired donors, with incorporation or reduction of molecular oxygen		ABE	0.3942
GO:0016701	oxidoreductase activity, acting on single donors with incorporation of molecular oxygen		ABE	0.3942
GO:0016721	oxidoreductase activity, acting on superoxide radicals as acceptor		ABE	0.4038
GO:0004096	catalase activity		ABE	0.4038
GO:0016886	ligase activity, forming phosphoric ester bonds		ABE	0.4135
GO:0019213	deacetylase activity		ABE	0.4135
GO:0005319	lipid transporter activity		ABE	0.4135
GO:0016725	oxidoreductase activity, acting on CH or CH2 groups		ABE	0.4327
GO:1901682	sulfur compound transmembrane transporter activity		ABE	0.4423
GO:0016730	oxidoreductase activity, acting on iron-sulfur proteins as donors		ABE	0.4423
GO:0016801	hydrolase activity, acting on ether bonds		ABE	0.4423
GO:0051002	ligase activity, forming nitrogen-metal bonds		ABE	0.4423
GO:0016992	lipoate synthase activity		ABE	0.4519
GO:0004076	biotin synthase activity		ABE	0.4519
GO:0016849	phosphorus-oxygen lyase activity		BE	0.4519
GO:0004134	4-alpha-glucanotransferase activity		ABE	0.4615
GO:0016872	intramolecular lyase activity		ABE	0.4712
GO:0004135	amylo-alpha-1,6-glucosidase activity		ABE	0.4808
GO:0016785	transferase activity, transferring selenium-containing groups		B	0.4904
GO:0046992	oxidoreductase activity, acting on X-H and Y-H to form an X-Y bond		BE	0.5
GO:0030247	polysaccharide binding		BE	0.5096
GO:0042030	ATPase inhibitor activity		B	0.5192
GO:0018836	alkylmercury lyase activity		B	0.5192
GO:0018706	pyrogallol hydroxytransferase activity		B	0.5192
GO:0046508	hydrolase activity, acting on carbon-sulfur bonds		B	0.5192
GO:0015066	alpha-amylase inhibitor activity		B	0.5192
GO:0016692	NADH peroxidase activity		AB	0.5192
GO:0010011	auxin binding		E	0.5288
GO:0016688	L-ascorbate peroxidase activity		E	0.5288
GO:0005325	peroxisomal fatty-acyl-CoA transporter activity		E	0.5385
GO:0009037	tyrosine-based site-specific recombinase activity		AB	0.5385
GO:0016826	hydrolase activity, acting on acid sulfur-nitrogen bonds		E	0.5385
GO:0016852	sirohydrochlorin cobaltochelatase activity		AB	0.5385
GO:0008036	diuretic hormone receptor activity		E	0.5385
GO:0030197	extracellular matrix constituent, lubricant activity		E	0.5481
GO:0015136	sialic acid transmembrane transporter activity		B	0.5481
GO:0005030	neurotrophin receptor activity		E	0.5481
GO:0030020	extracellular matrix structural constituent conferring tensile strength		E	0.5481
GO:0015169	glycerol-3-phosphate transmembrane transporter activity		B	0.5481
GO:0016691	chloride peroxidase activity		B	0.5577
GO:0016824	hydrolase activity, acting on acid halide bonds		ABE	0.5577
GO:0016989	sigma factor antagonist activity		B	0.5673
GO:0008379	thioredoxin peroxidase activity		BE	0.5673
GO:0010577	metalloenzyme activator activity		E	0.5673
GO:0016532	superoxide dismutase copper chaperone activity		E	0.5673
GO:0015232	heme transporter activity		ABE	0.5673
GO:0047485	protein N-terminus binding		E	0.5673
GO:0001512	dihydronicotinamide riboside quinone reductase activity		BE	0.5673
GO:0015119	hexose phosphate transmembrane transporter activity		E	0.5673
GO:0030882	lipid antigen binding		E	0.5673
GO:0004130	cytochrome-c peroxidase activity		ABE	0.5769
GO:0015039	NADPH-adrenodoxin reductase activity		E	0.5769
GO:0016661	oxidoreductase activity, acting on other nitrogenous compounds as donors		ABE	0.5769
GO:0030549	acetylcholine receptor activator activity		E	0.5962
GO:0008665	2'-phosphotransferase activity		E	0.6058
GO:0008398	sterol 14-demethylase activity		BE	0.6058
GO:0051185	coenzyme transporter activity		E	0.6058
GO:0000170	sphingosine hydroxylase activity		E	0.6058
GO:0019826	oxygen sensor activity		E	0.6058
GO:0008434	calcitriol receptor activity		E	0.6154
GO:0005308	creatine transmembrane transporter activity		E	0.6154
GO:0001918	farnesylated protein binding		E	0.6154
GO:0031626	beta-endorphin binding		E	0.625
GO:0030021	extracellular matrix structural constituent conferring compression resistance		E	0.625
GO:0030550	acetylcholine receptor inhibitor activity		E	0.625
GO:0019782	ISG15 activating enzyme activity		E	0.625
GO:0010852	cyclase inhibitor activity		E	0.625
GO:0031491	nucleosome binding		E	0.6346
GO:0030977	taurine binding		E	0.6346
GO:0042393	histone binding		E	0.6346
GO:0030492	hemoglobin binding		E	0.6346
GO:0019809	spermidine binding		E	0.6442
GO:1901702	salt transmembrane transporter activity		E	0.6442
GO:0004952	dopamine neurotransmitter receptor activity		E	0.6442
GO:0017129	triglyceride binding		E	0.6442
GO:0004447	iodide peroxidase activity		E	0.6538
GO:0003826	alpha-ketoacid dehydrogenase activity		E	0.6538
GO:0016176	superoxide-generating NADPH oxidase activator activity		E	0.6635
GO:0060229	lipase activator activity		E	0.6635
GO:0019211	phosphatase activator activity		E	0.6635
GO:0055102	lipase inhibitor activity		E	0.6635
GO:0070643	vitamin D 25-hydroxylase activity		E	0.6731
GO:0002054	nucleobase binding		E	0.6731
GO:0019855	calcium channel inhibitor activity		BE	0.6731
GO:0004873	asialoglycoprotein receptor activity		E	0.6731
GO:0004998	transferrin receptor activity		E	0.6731
GO:0030159	receptor signaling complex scaffold activity		E	0.6827
GO:0005328	neurotransmitter:sodium symporter activity		E	0.6827
GO:0030881	beta-2-microglobulin binding		E	0.6827
GO:0019871	sodium channel inhibitor activity		BE	0.6923
GO:0030023	extracellular matrix constituent conferring elasticity		E	0.6923
GO:0008200	ion channel inhibitor activity		BE	0.6923
GO:0015229	L-ascorbic acid transporter activity		E	0.6923
GO:0008316	structural constituent of vitelline membrane		E	0.6923
GO:0003708	retinoic acid receptor activity		E	0.6923
GO:0010698	acetyltransferase activator activity		E	0.7019
GO:0090482	vitamin transmembrane transporter activity		E	0.7019
GO:0005521	lamin binding		E	0.7019
GO:0010521	telomerase inhibitor activity		E	0.7019
GO:0016936	galactoside binding		E	0.7115
GO:0015026	coreceptor activity		E	0.7115
GO:0019212	phosphatase inhibitor activity		E	0.7115
GO:0005522	profilin binding		E	0.7115
GO:0031210	phosphatidylcholine binding		E	0.7115
GO:0004000	adenosine deaminase activity		BE	0.7115
GO:0005550	pheromone binding		E	0.7212
GO:0008656	cysteine-type endopeptidase activator activity involved in apoptotic process		E	0.7212
GO:0003923	GPI-anchor transamidase activity		E	0.7212
GO:0019992	diacylglycerol binding		E	0.7212
GO:0016504	peptidase activator activity		E	0.7212
GO:0008073	ornithine decarboxylase inhibitor activity		E	0.7212
GO:0004016	adenylate cyclase activity		BE	0.7212
GO:0031072	heat shock protein binding		E	0.7212
GO:0070576	vitamin D 24-hydroxylase activity		E	0.7212
GO:0015197	peptide transporter activity		BE	0.7212
GO:0005527	macrolide binding		E	0.7308
GO:0030742	GTP-dependent protein binding		E	0.7308
GO:0031369	translation initiation factor binding		E	0.7308
GO:0015144	carbohydrate transmembrane transporter activity		ABE	0.7308
GO:0004800	thyroxine 5'-deiodinase activity		BE	0.7308
GO:0000900	translation repressor activity, nucleic acid binding		E	0.7308
GO:0016822	hydrolase activity, acting on acid carbon-carbon bonds		ABE	0.7308
GO:0072349	modified amino acid transmembrane transporter activity		E	0.7404
GO:0000149	SNARE binding		E	0.7404
GO:0005220	inositol 1,4,5-trisphosphate-sensitive calcium-release channel activity		E	0.7404
GO:0005338	nucleotide-sugar transmembrane transporter activity		E	0.7404
GO:0030332	cyclin binding		E	0.7404
GO:0019209	kinase activator activity		E	0.7404
GO:0051219	phosphoprotein binding		E	0.75
GO:0010861	thyroid hormone receptor activator activity		E	0.75
GO:0019781	NEDD8 activating enzyme activity		E	0.75
GO:0008494	translation activator activity		E	0.75
GO:0017147	Wnt-protein binding		E	0.7596
GO:0008559	xenobiotic-transporting ATPase activity		ABE	0.7596
GO:0008329	signaling pattern recognition receptor activity		E	0.7596
GO:0030249	guanylate cyclase regulator activity		E	0.7596
GO:0004887	thyroid hormone receptor activity		E	0.7596
GO:0005346	purine ribonucleotide transmembrane transporter activity		E	0.7692
GO:0030228	lipoprotein particle receptor activity		E	0.7692
GO:0030250	guanylate cyclase activator activity		E	0.7692
GO:0010853	cyclase activator activity		E	0.7692
GO:0002039	p53 binding		E	0.7788
GO:0005337	nucleoside transmembrane transporter activity		E	0.7788
GO:0019840	isoprenoid binding		E	0.7885
GO:0005047	signal recognition particle binding		E	0.7981
GO:0019838	growth factor binding		E	0.7981
GO:0098599	palmitoyl hydrolase activity		E	0.8077
GO:0008061	chitin binding		ABE	0.8077
GO:0008565	protein transporter activity		BE	0.8077
GO:0016722	oxidoreductase activity, oxidizing metal ions		ABE	0.8077
GO:0009881	photoreceptor activity		ABE	0.8077
GO:0060590	ATPase regulator activity		ABE	0.8173
GO:0047623	adenosine-phosphate deaminase activity		E	0.8173
GO:0048551	metalloenzyme inhibitor activity		BE	0.8269
GO:0003707	steroid hormone receptor activity		BE	0.8269
GO:0098531	direct ligand regulated sequence-specific DNA binding transcription factor activity		BE	0.8269
GO:0008013	beta-catenin binding		E	0.8269
GO:0008022	protein C-terminus binding		E	0.8365
GO:0030674	protein binding, bridging		E	0.8365
GO:0035591	signaling adaptor activity		E	0.8365
GO:0050839	cell adhesion molecule binding		E	0.8365
GO:0008134	transcription factor binding		E	0.8462
GO:0001965	G-protein alpha-subunit binding		E	0.8462
GO:0000981	sequence-specific DNA binding RNA polymerase II transcription factor activity		BE	0.8462
GO:0036459	ubiquitinyl hydrolase activity		E	0.8462
GO:0042277	peptide binding		E	0.8462
GO:0019787	ubiquitin-like protein transferase activity		E	0.8462
GO:0019210	kinase inhibitor activity		E	0.8462
GO:0030594	neurotransmitter receptor activity		E	0.8558
GO:0008092	cytoskeletal protein binding		E	0.8654
GO:0005044	scavenger receptor activity		BE	0.875
GO:0019904	protein domain specific binding		E	0.8942
GO:0001653	peptide receptor activity		E	0.8942
GO:0022803	passive transmembrane transporter activity		BE	0.9038
GO:0032403	protein complex binding		E	0.9038
GO:0005539	glycosaminoglycan binding		BE	0.9038
GO:0061135	endopeptidase regulator activity		ABE	0.9231
GO:0030414	peptidase inhibitor activity		ABE	0.9231
GO:0005516	calmodulin binding		BE	0.9327
GO:0004888	transmembrane signaling receptor activity		ABE	0.9327
GO:0005102	receptor binding		BE	0.9327
GO:0005543	phospholipid binding		BE	0.9423
GO:0019887	protein kinase regulator activity		BE	0.9615
GO:0019888	protein phosphatase regulator activity		E	0.9712
GO:0019899	enzyme binding		E	0.9712
GO:0016229	steroid dehydrogenase activity		ABE	0.9712
GO:0005484	SNAP receptor activity		BE	0.9712
GO:0001076	RNA polymerase II transcription factor binding transcription factor activity		E	0.9808
GO:0005344	oxygen transporter activity		ABE	0.9904
GO:0005496	steroid binding		ABE	0.9904
GO:0003712	transcription cofactor activity		E	0.9904
GO:0043178	alcohol binding		ABE	0.9904
GO:0030695	GTPase regulator activity		E	1
GO:0004602	glutathione peroxidase activity		BE	1
GO:0016790	thiolester hydrolase activity		E	1
GO:0048029	monosaccharide binding		BE	1

**Fig 12:**
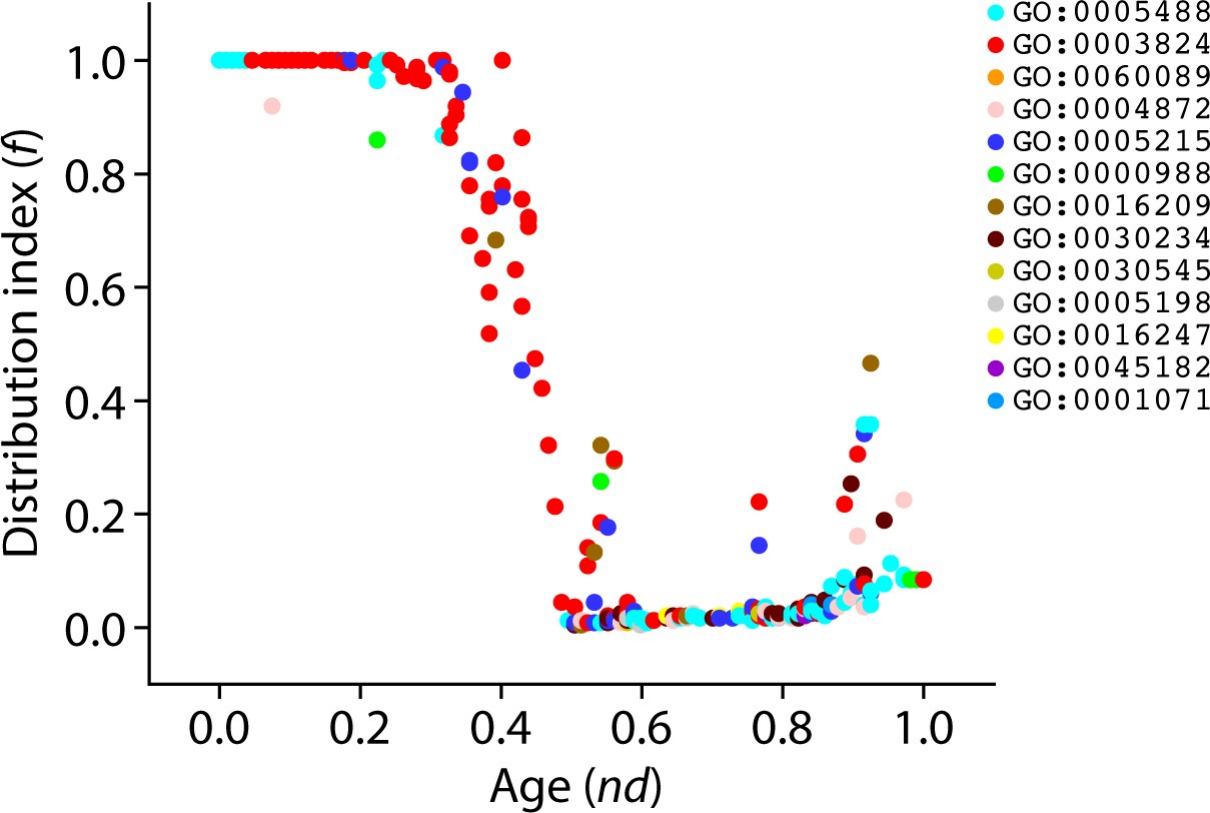
Order of the evolutionary appearence of level 3 GO terms. Scatter plot highlighting the distribution of level 3 GO terms with respect to age (*nd*) and molecular functions are colored according to corresponding level 1 GO terms.

Again, to unfold the data carried in the ToF, we calculated the distribution index *f* of level 3 GO terms (Fig 12). The most ancient level 3 GO terms were universally present in organisms (*f* = 1) and their representation decreased along with increasing age. Interestingly, we observed a pattern of increase in *f* values manifesting quite late in evolution (*nd* > 0.74). This same trend was observed for level 2 GO terms (Fig 10). These findings support the expected initial decrease in the spread of functional toolkits (*f* < 1) induced by the diversification of functionomes in individual lineages, which is then counteracted by a pattern of increased spread of novel functions (*f* > 0) occurring late in evolution via recruitment and horizontal exchange.

**Fig 13:**
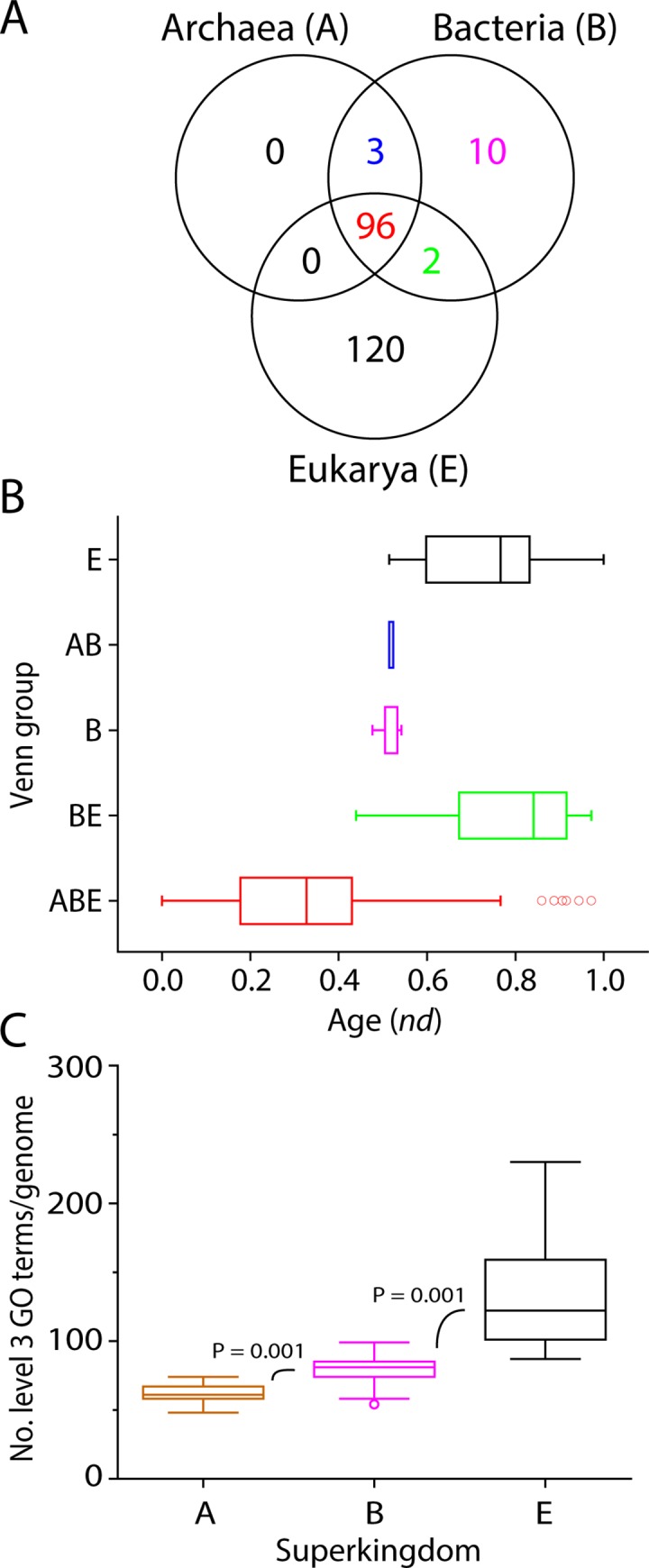
The distribution of level 3 molecular functions in life. (A) The Venn diagram illustrates the distribution of level 3 GO terms in the three superkingdoms. (B) Boxplots show the age of level 3 GO terms in superkingdoms. (C) Boxplots shows the number of level 3 GO terms per genome in superkingdoms. Comparisons are significant at 0.001 level. A: Archaea, B: Bacteria, E: Eukarya.

Mapping Venn taxonomic groups in the timeline of level 3 molecular functions indicated that the ABE taxonomic group appeared first and the age of its members ranged widely, from *nd* = 0~1 (Fig 13A and 13B). Its appearance was followed by the appearance of BE, B, AB, and E in that order (Fig 13B). The first GO terms of the BE, B and AB Venn groups were GO:0016849 (phosphorus-oxygen lyase activity), GO:0016785 (transferase activity, transferring selenium-containing groups) and GO:0016692 (NADH peroxidase activity), respectively. On the other hand, the first E-specific functions were GO:0016688 (L-ascorbate peroxidase activity) and GO:0010011 (auxin binding). At this level, Bacteria-specific functions appeared in general earlier than those of other superkingdom-specific groups. Similar to level 2 molecular functions (Fig 6), Eukarya-specific level 3 molecular functions were higher in number than even those of the common ABE Venn group indicating massive functional eukaryotic diversity (Fig 13A). Boxplots describing the genomic diversity of level 3 GO terms showed it ranged 48–74 for Archaea, 54–99 for Bacteria, and 87–230 for Eukarya (Fig 13C). The massive number of unique Eukaryal molecular functions is important considering we sampled only 21 Eukaryal genomes. Several studies suggested that gene duplications and rearrangements were abundant during evolution of eukaryotes and contributed significantly to the tailoring of eukaryotic genomes [21, 36, 37]. It is therefore likely that these events caused the relatively rapid functional diversification of ancient molecular functions we reveal in our timelines, which suggest the generation of novel functional profiles with more specialized functions in Eukarya than in other superkingdoms. In contrast, Archaea and Bacteria favor a strategy of economy by developing functional activities that were simpler [21].

### General patterns of evolutionary accumulation of GO terms

**Fig 14:**
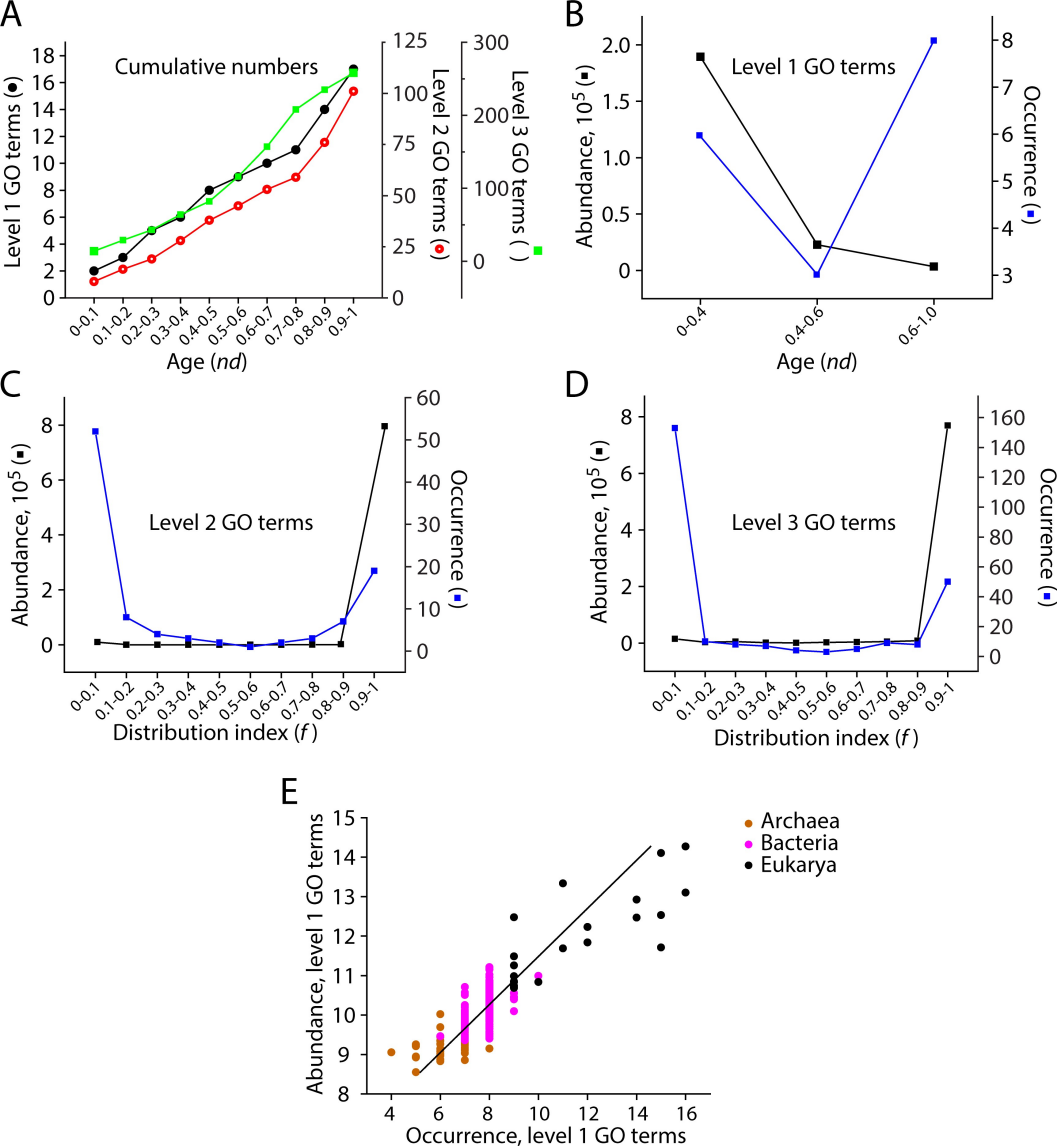
Relationship between abundance and occurrence. (A) Accumulation of level 1, level 2 and level 3 molecular functions along the evolutionary timeline. (B) Plot of abundance and occurence for level 1 GO terms against their age (*nd*) describes diversity of level 1 of DAG_mf_. (C) Plot of abundance and occurence for level 2 GO terms against their age (*nd*). (D) Plot of abundance and occurence for level 3 GO terms against their age (*nd*). (E) Functional diversity (occurence) plotted against the abundance of level 1 GO terms for 249 proteomes. The three superkingdoms are colored. Abundance values are provided in log2 scale.

We observed that the accumulation of level 1, level 2 and level 3 GO terms in the timeline were significantly and similarly correlated (y = 6.13x – 8.01; *R*^*2*^ = 0.98 for level 2 and y = 18.25x – 34; *R*^*2*^ = 0.94 for level 3) (Fig 14A). Several evolutionary forces lead to the accumulation of functions in genomic repertoires, including HGT, gene rearrangements, and gene duplications [8, 14, 26]. These events shape and increase genomic abundance and corresponding molecular functions [8, 42, 47]. Therefore, abundance is a naturally occuring biological process that is very valuable for phylogeny reconstruction [7, 8, 21, 42]. While occurence based analyses provide non-redundant representations of genes and their functions and usually result in more balanced ToL topologies [8, 53], we observed that abundance and occurence of GO terms expressed contrasting relationships (Fig 14B, 14C and 14D). For instance, plotting abundance and occurence of level 1 GO terms in the evolutionary timeline (*nd*) revealed abundance was highest at 0 ≤ *nd* < 0.4 when relatively ancient level 1 GO toolkits were developing (Fig 13B). In contrast, the abundance of level 1 GO terms substantially decreased later on in evolution at 0.4 ≤ *nd* <0.6, when lower numbers of new functions were acquired. Finally, the booming of new functions was observed at a later stage, 0.6 ≤ *nd* < 1, with new functions exhibiting low abundance values (Fig 14B). This low abundance could be related to new functions that had not enough time to spread in different organisms and increase in individual genomic repertoires. They also correspond to taxon-specific functions that developed late in evolution (Fig 2). When plotting genomic abundance and occurence against the distribution index *f* at level 2 and level 3 of molecular functions, the majority of functions (~55 or >50% level 2 and ~160 or >50% for level 3) were not conserved across taxa (*f* < 0.1) but were spread with low abundance value (~2,900/functionome for level 2 and ~14,000/functionome for level 3) (Fig 14C and 14D). Similar to the new functional toolkit of level 1 of the DAG_mf_, these terms also represent relatively new molecular functions that have been gained by functionomes late in evolution. Moreover, the graphs of level 2 and level 3 GO terms display biphasic pattern of distribution. These biphasic patterns first decreased and then started to increase at 0.9 <*f* < 0.1. These GO terms that were universal to superkingdoms were low in number (~ 20 for level 2 and ~ 50 for level 3) and held the highest abundance values (~ 790,000 for level 2 and ~ 770,000 for level 3). Universal GO terms are crucial for cellular life and conserved across most taxa (e.g., ion binding, cofactor binding, signal transducer activity for level 2 terms; anion binding, nucleotide binding, coenzyme binding for level 3 GO terms). A plot that describes the relationships between diversity and abundance of level 1 GO terms reveals that Archaea has the simplest functionomes followed by Bacteria and Eukarya, in that order (Fig 14E) with high correlation (y = 0.44x + 6.75; *R*^*2*^ = 0.72). Organisms follow a congruent trend towards functional diversity and organismal complexity, which matches inferences from the appearance of Venn groups in the timelines. This trend also confirms our initial evolutionary model of proteome growth [30, 54].

### Surveying the functional makeup of functionomes accross the GO hierarchy

**Fig 15:**
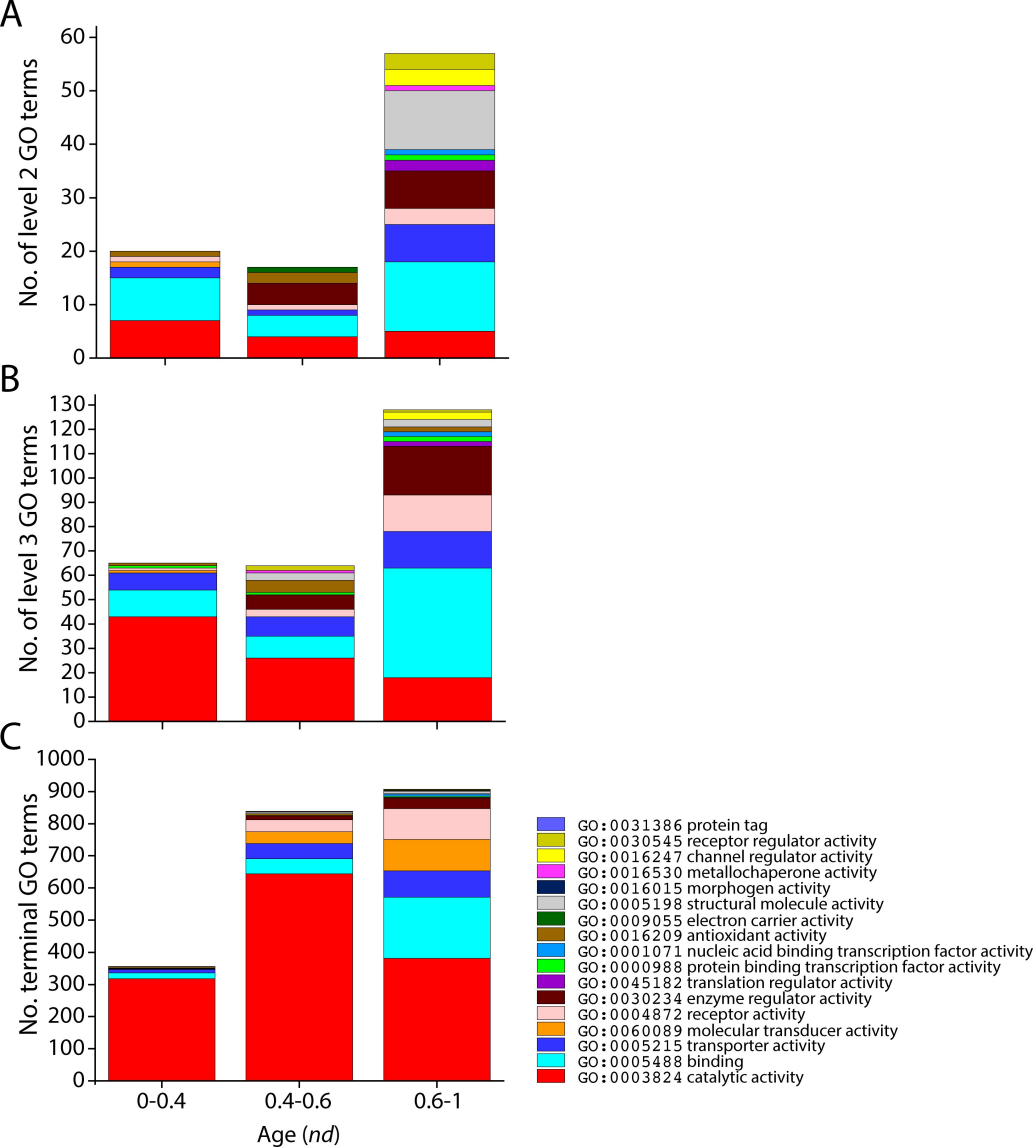
Functional disribution of GO terms at various levels in level 1 GO functional categories. (A) Histograms comparing the number of level 2 GO terms corresponding to each level 1 GO of DAG_mf_. (B) Histograms comparing the number of level 3 GO terms corresponding to each level 1 GO of DAG_mf_. (C) Histograms comparing the number of terminal GO terms corresponding to each level 1 GO of DAG_mf_.

We studied the molecular functions of level 2, level 3 and terminal GO terms present in the 249 functionomes analyzed using functional annotations corresponding to level 1 GO terms of the DAG_mf_. When plotted against three age bins, a drop is seen in the middle 0.4–0.6 *nd* range for level 2 GO terms (17) (Fig 15A). This bin includes a period marked by massive gene loss in cellular organisms and even viruses [54]. The majority of level 2 GO terms (57) occurred late (0.6 < *nd* ≤ 1) in a period encompassing superkingdom diversification and genome expansion in Eukarya [30, 54]. Most of the very early (*nd* < 0.4) level 2 GO terms held catalytic activity and binding functions, suggesting the primacy of metabolism and cofactor binding in metabolic enzymes. An increasing trend for catalytic activity in the timeline suggests that after massive gene loss cellular organisms increased catalytic activity, binding function, enzyme regulatory activity, and transporter activity in order to adapt to new lifestyles. These functions involve ion binding, small molecule binding, transferase activity, oxidoreductase activity and so on. In contrast, we see a relatively even distribution of level 3 GO terms in the early 0–0.4 and middle 0.4–0.6 *nd* periods (Fig 15B). The majority of functionomes defined at level 3 of the DAG_mf_ unfolded very late (128 in the 0.6–1 *nd* range). Remarkably, catalytic activity was the most abundant function of the first two age bins while binding was the most popular function (45) in the 0.6–1 *nd* range (Fig 15B). We also observed that the majority of functionomic makeup defined with terminal GO terms, the most modern definition of function, appeared quite late (838 and 907 terms for the 0.4–0.6 and 0.6–1 *nd* periods, respectively) (Fig 15C). Interestingly and contrary to other hiearchical levels, the distribution of terminal GO terms was not higher in the early 0–0.4 and increased in the middle 0.4–0.6 age bin. Furthermore, catalytic activity was the most popular activity thoughout the timeline (1343 terminal terms) followed by binding (255 terms) and transporter activity (140 terms). At terminal GO level, cellular organisms acquired the most specialized functions, which were needed for the specific organismal lifestyles of an expanding cellular world [21, 54].

**Fig 16:**
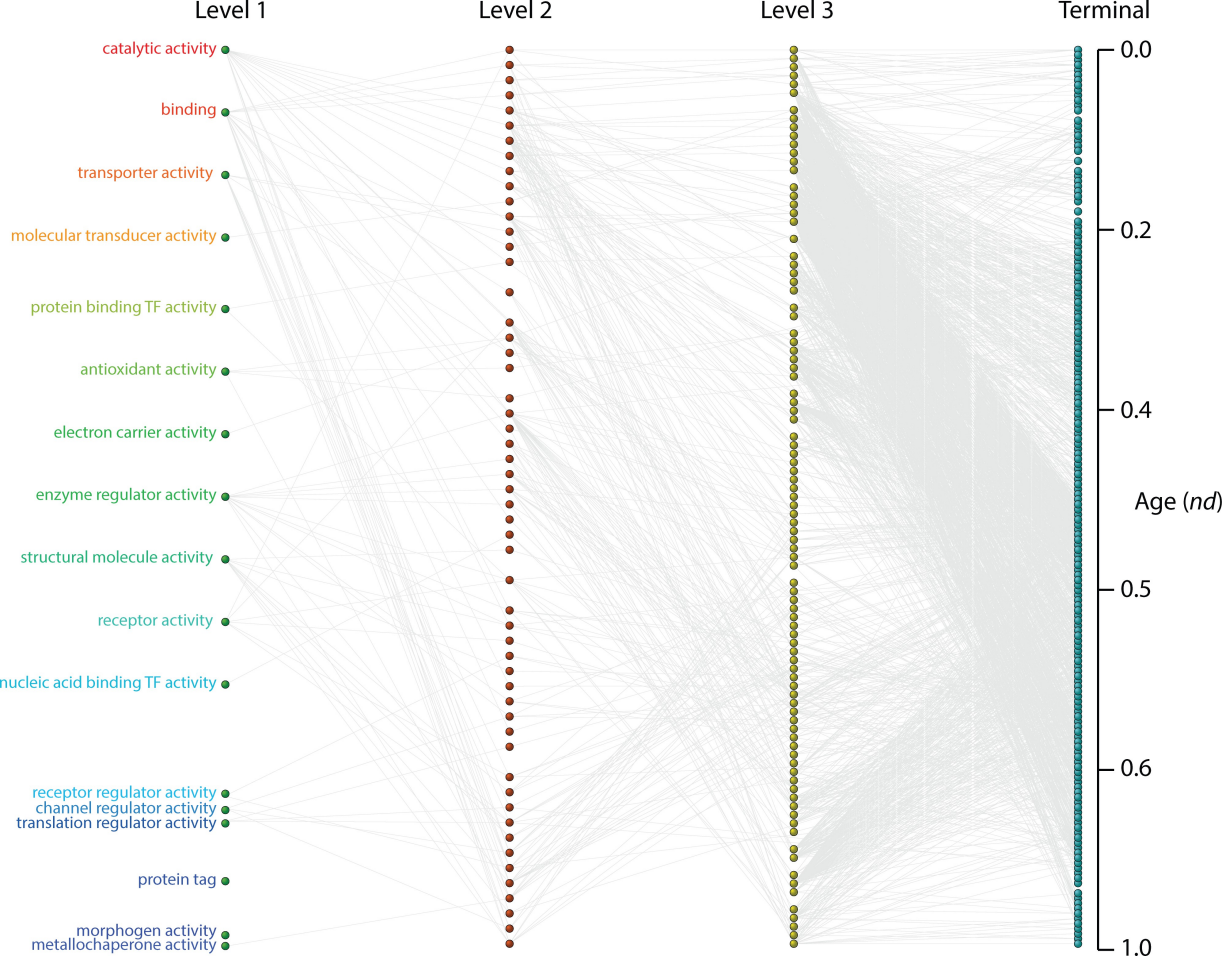
A gene ontology network inferred from age (*nd*) and hierarchy of terms. The diagram shows a large networks of molecular functions and their interrelationships. The GO terms were sorted by *nd* value in the y axis. Green circles: level 1 (17 GO terms); orange circles: level 2 (101 GO terms); yellow circles: level 3 (257 GO terms) and, cyan circles: terminal GO terms (1891 GO terms). Many GO terms overlapped in the network. The network was created with the Pajek program.

Network analyses revealed an hourglass pattern of enzyme recruitment in the molecular functions of the DAG (Fig 16). Old functions were thoroughly used in younger lower level alternatives and very young ones throughout the timeline. This pattern repeated in every one of the three mappings, which gives a criss-cross pattern. Catalytic activity had connections with level 2 GO terms throughout the timeline. On the other hand, binding function had connections with ancient GO terms and relatively new GO terms. Interestingly, we see hub GO terms (Tables 5 and 6) that mostly map to catalytic activity at level 2 and level 3 suggesting metabolic activity dominated the network throughout history. Remarkably, all six enzymatic activities (transferase, hydrolase, oxidoreductase, ligase, lyase and isomerase activity) were present in the network as hubs. Moreover, a bipashic pattern is obvious in the networks starting with enzyme regulator activity, a result that matches CA analyses (Fig 3A) as enzyme regulator activity has only connections with relatively new level 2 terms (*nd* ~ 0.42) with one exception for receptor activity which matches a connection with an older function (*nd* ~ 0.3).

**Table 5:** Hub level 2 GO terms linked to level 3 GO terms sorted by *nd* value.

Level 2 GO terms	Link number	Annotation	*nd*
GO:0097159	7	organic cyclic compound binding	0.01
GO:1901363	6	heterocyclic compound binding	0.01
GO:0036094	6	small molecule binding	0.03
GO:0016740	11	transferase activity	0.07
GO:0016787	16	hydrolase activity	0.08
GO:0016491	30	oxidoreductase activity	0.10
GO:0016874	6	ligase activity	0.12
GO:0016829	7	lyase activity	0.13
GO:0016853	6	isomerase activity	0.15
GO:0022857	19	transmembrane transporter activity	0.19
GO:0038023	15	signaling receptor activity	0.31
GO:0004601	8	peroxidase activity	0.32
GO:0005515	29	protein binding	0.41
GO:0008289	6	lipid binding	0.44
GO:0008047	9	enzyme activator activity	0.47
GO:0004857	9	enzyme inhibitor activity	0.49
GO:1901505	6	carbohydrate derivative transporter activity	0.93

**Table 6:** Hub level 3 GO terms linked to terminal GO terms sorted by *nd* value.

Level 3 GO terms	Link number	Annotation	*nd*
GO:0016772	163	transferase activity, transferring phosphorus-containing groups	0.047
GO:0038023	127	signaling receptor activity	0.065
GO:0016817	60	hydrolase activity, acting on acid anhydrides	0.075
GO:0016788	106	hydrolase activity, acting on ester bonds	0.084
GO:0016614	78	oxidoreductase activity, acting on CH-OH group of donors	0.093
GO:0016879	34	ligase activity, forming carbon-nitrogen bonds	0.112
GO:0016741	58	transferase activity, transferring one-carbon groups	0.121
GO:0016830	57	carbon-carbon lyase activity	0.131
GO:0016810	59	hydrolase activity, acting on carbon-nitrogen (but not peptide) bonds	0.149
GO:0016757	81	transferase activity, transferring glycosyl groups	0.159
GO:0016835	39	carbon-oxygen lyase activity	0.159
GO:0016746	62	transferase activity, transferring acyl groups	0.168
GO:0022891	121	substrate-specific transmembrane transporter activity	0.177
GO:0022804	73	active transmembrane transporter activity	0.187
GO:0016820	32	hydrolase activity, acting on acid anhydrides, catalyzing transmembrane movement of substances	0.187
GO:0016627	32	oxidoreductase activity, acting on the CH-CH group of donors	0.206
GO:0016798	56	hydrolase activity, acting on glycosyl bonds	0.336
GO:0004497	53	monooxygenase activity	0.383
GO:0016705	72	oxidoreductase activity, acting on paired donors, with incorporation or reduction of molecular oxygen	0.383
GO:0051213	35	dioxygenase activity	0.383
GO:0001653	30	peptide receptor activity	0.916
GO:0022803	30	passive transmembrane transporter activity	0.925
GO:0004888	102	transmembrane signaling receptor activity	0.972
GO:0005102	93	receptor binding	0.972

## Discussion

### ToFs and the use of functionomes as phylogenetic characters

The main goal of our study was to track the evolutionary history of molecular functions defined at different levels of the DAG_mf_ using an updated and expanded functional dataset. The GOA Project provides reliable information of gene products by manually curating and validating electronic annotations. We downloaded GO terms from GOA datasets to create data matrices with molecular functions as rows and functionomes as columns. These matrices were used to reconstruct rooted ToFs describing the origin and evolution of molecular functions at different levels of the GO hierarchy. Each ToF holds molecular functions (suitably defined at ontological level) as taxa (the leaves of the tree) and defines phylogenetic relationships (tree topologies) based on their abundance in the functionomes analyzed (the phylogenetic characters and data analyzed). We stresss that phylogenetic signal rests in the sets of characters that are used to reconstruct phylogenies, i.e. the collectives of gene products that are part of genomes. Because function is more conserved that molecular sequence and sometimes more conserved than 3D structure, historical relationships are evolutionarily deep and can inform about the history of the functionomes of organisms. In general, the sequence of the small subunit of rRNA has been widely used as a gold standard for building ToLs despite of it representing only one of the three major rRNA subunits and only one of dozens of ribosomal molecules [8]. The finding that rRNA coevolves with ribosomal proteins and that rRNA is younger than tRNA complicates even further the arguments favoring the evolutionary centrality of only one component of the ribosome [8, 55]. In contrast, our dataset makes use of a collective of functionomes and their functional components summarizing the biochemical and cellular behavior of most macromolecules of the cell [56]. ToLs describing the evolution of functionomes were more reliable in displaying monophyletic relationships than those built from rRNA [8]. In light of these considerations, our approach provides a more powerful alternative to canonical historical reconstruction methods that focus on small subsets of macromolecules.

Biological functions manifest as molecular activities through the interaction of parts of molecules and in the contextual environments of cells and organisms [17]. Molecular functions also interact with each other and with the external environment through genomic attributes such as the genetic quest to increase organismal fitness. In this study, GO terms provide a structured and controlled vocabulary (of occurrents and continuants) with a very descriptive meaning of biology. Their use as taxa allows tracing back their origin and evolution in living systems. In order to integrate ToFs generated at different levels of the GO hierarchy, we hypothesize that modern functions somehow carry the remnants of ancestral functions by unbroken chains of lineage, and that these chains express themselves differently according to the level of GO abstraction. This notion can be rationalized because molecular functions are the manisfestation of the evolving biology of an organism, which holds a collective of different histories in their molecular component parts [7, 17, 57]. This pluralistic historical connection can be retrodicted by mining the hierarchical network diagram of GO terms and using these terms as phylogenetic characters to study the evolution of molecular functions in functionomes, and indirectly, organisms. Here we expand a previous evolutionary study of ontological terms in the functionomes of 38 bacterial and eukaryal organisms [17] to 249 organisms representing the three superkingdoms of life. We followed a phylogenomic maximum parsimony strategy that uses abundance of GO terms as ordered multistate characters to reconstruct ToFs at different levels of the DAG_mf_. The algorithmic searches of most parsimonious trees are robust, approach maximum likelihood estimations, decrease the number of *ad hoc* assumptions, and use a minimum number of auxiliary assumptions (e.g. rooting does not require definition of outgroup taxa).

The mapping of lower-level functions to higher-level functions in the DAG_mf_ is tangle-like and complex (S2 Fig). For example, one terminal GO term can have several parent GO terms and these parents of terminal terms can appear at different levels of the GO hierarchy. This complicates the mapping of corresponding parent to terminal GO terms and the interpretation of taxa. While phylogenetic GO characters follow the tenets of transformational homology [58], GO terms at lower level have more specialized functional annotations and also connections with its parent(s) enabling better retrodictive views of functional evolution.

We used the number of GO terms in a genome (genomic abundance) as a phylogenetic linearly ordered multistate character. This number can increase or decrease in any branch of a ToF and consequently in the evolutionary timeline derived from it. While the leaves of the ToF are GO terms, the tree that we build from the data does not arise from a model of change that involves structural or ontological transformations of molecular functions. Instead, the historical relationships of GO terms are inferred directly from quantitative information in genomic makeup. This ‘criterion of primary homology’ rests exclusively on genomic abundance of individual GO terms (functionome make up), and its validity is permanently tested by mutual optimization of phylogenetic signal in characters and tree reconstruction (an exercise known as Hennigian illumination). Thus, the methodology operates under the hypothetico-deductive framework of empirical content of theories and degree of corroboration. We note that the historical signal and reliability of ToFs that have been published are now being strengthened by more data and improved annotations (e.g. more functionomes and better sampling of the world of organisms and viruses).

ToFs are rooted *a posteriori* with a criterion of transformational homology that is popular in systematic biology studies. The polarization of linearly ordered (additive) character state transformations typical of Wagner parsimony is used to root most parsimonious unrooted trees retained after heuristic searches of tree space. Polarization of ToFs complies with Weston’s generality rooting criterion, in which the taxic distribution of a character state is a subset of the distribution of another. This criterion stresses homology and additive phylogenetic change in the nested pattern of a phylogeny, and can be induced by a process model of change in which molecular function grows in abundance by accumulation of functional variants. This assumption has been succesfully applied to molecules in the reconstruction of phylogenetic trees of protein domain structure and molecular functions [8, 14, 17, 30, 36, 42]. With Lundberg rooting, however, the polarization criterion is not *ad hoc* but empirically tested *a posteriori* by empirically confirming which of the two polarization directions is optimal. Operationally, a GO term that is more abundant will tend to appear at the base of the ToF if the tree was reconstructed using a single functionome as phylogenetic character (representing a single organism). This term will tend to remain at the base if it is widely distributed in different functionomes. In addition, shared and derived changes in the abundance of the term can also strengthen the basal position of the taxon. The larger the number of characters (functionomes) the more robust the basal placement of the taxon.

### ToFs and the onset of organismal diversification

The timelines of molecular functions derived from ToFs showed congruent evolutionary patterns of origin and diversification of the functionomes of superkindoms. The most ancient functions along the timelines were either universal or widely distributed in the functionomes of organisms (their distribution index *f* approached 1). They included 8 molecular functions at level 1, 36 molecular functions at level 2, and 76 molecular functions at level 3 of the DAG_mf_. The tendency of increase in the number of shared molecular functions likely portrays the evolutionary complexity of early life defined with increasingly more modern definitions. These universal functions define the functional toolkit that was present in the “rich communal world” of the urancestor of life [30]. During that time, it is likely that these functions exchanged widely between cells in processes analogous to those of modern HGT, even before significant barriers to information exchange were established [30, 59]. It is also possible that ancient functions were retained because of their central importance to the workings of the emergent cells, giving rise to proper functional subsets that now describe the metabolic activities of modern cells. A substantial 47% of level 2 GO terms and 29% of level 3 GO terms were part of the ABE Venn taxonomic group. Furthermore, at *nd* < 0.2, level 2 molecular functions were present in all proteomes (*f* = 1) and level 3 functions in amost all proteomes (*f* ~ 0.92). These observations confirm the centrality of an ancient universal biology operating at the molecular function level. The distribution of level 2 GO terms in Archaea was remarkably similar to Bacteria, where most of the ancient functions were spread with high *f*. In Archaea and Bacteria, 17 and 21 level 2 GO terms of ancient origin (*nd* < 0.41) were universally present in each superkingdoms (*f =* 1), respectively. Conversely, Eukarya had 29 level 2 GO terms (*nd* < 0.64) that were universally present in eukaryal organisms. In general, these unifying patterns dominated the first half of the evolutionary timeline. During the second half, molecular functions became less distributed in organisms, with *f* values decreasing with increasing *nd* (Fig 4). This is an expected outcome from the selective gain and/or loss of molecular functions in the increasingly diversifying lineages of the ToL. Several factors affect genomic abundance and explain changes in *f*, including vertical inheritance, genome reduction or expansion, HGT, and recruitment processes [17, 21, 30]. These processes of divergence and convergence impact the discovery of functions and structures. For example, genome reduction can simplify the number of molecules and their associated functions in a superkingdom, while genome expansion can favor retaining functions, leading to decreases and increases of *f*, respectively [17]. These processes of gain and loss are pervasive in the timelines [44]. Early in evolution, genome reduction tendencies tend to lower *f* values even if molecular functions are old. Late in evolution, recruitment and other processes of horizontal transfer tend to increase *f* values even if molecular functions are young (Fig 4). For Archaea and Bacteria, *f* approached a minumum value at *nd* ~ 0.47 at a time when the first superkingdom-specific level 2 function, enzyme activator activity (GO:0008047), appeared in Eukarya (see Table 3 and Fig 10). Eukarya specific level 2 functions appeared relatively late at *nd* > 0.47 with very low *f* values (Fig 10). Their appearance coincided with the rise of a number of functions shared by Eukarya and Bacteria, all with very low *f* values. The sharp decline of *f* values of ABE functions immediately preceding E and EB functions in the timeline suggests either a strong reductive evolutionary model of functional loss for ancestors of all superkingdoms or wide recruitment in emerging lineages of diversified life. The only level 2 term shared by Archaea and Eukarya, GO:0030337–DNA polymerase processivity factor activity, appeared during that time at *nd* = 0.64. Similarly, the only two terms shared by Archaea and Bacteria, GO:0008907–integrase activity and GO:0009009–site-specific recombinase activity, appeared very late at *nd* = 1. Both molecular functions involve DNA cleavage and ligation during site-specific recombination [60]. Site-specific recombinases are widespread in Bacteria and Archaea as well as in certain yeast strains but in general they are not present in Eukarya [61]. These yeast strains probably gained the enzymes from Bacteria through HGT events.

Terminal GO terms mapped to 8, 12 and 17 level 1 molecular functions in Archaea, Bacteria and Eukarya, respectively (Fig 4). This simple observation reveals a progression of functional complexity that follows the order, Archaea, Bacteria and Eukarya. The order matches the rise of superkingdoms inferred from molecular structures [30] and functions [21]. It is unlikely that this progression is due to quality of GO data, since GO coverage for Archaea (57%) was not far away from that of Bacteria and Eukarya (60%) [21]. Remarkably, the timelines show Eukarya unfolding an extremely rich toolkit of functions when compared to the other superkingdoms. It is therefore likely that Eukarya retained most of the functional diversity of the urancestral stem line of descent and jump-started pathways of functional diversification much earlier than Bacteria and Archaea. This would explain the huge diversity of species and levels of organization that are present in the eukaryal superkingdom.

This vertical and ancient evolutionary trace was displayed by mapping level 1 GO terms to corresponding level 2 and level 3 terms (Figs 6 and 13) and sorting the mappings by Venn taxonomic groups (S3 Fig). A substantial number of level 2 and level 3 Eukarya-specific (E) GO terms mapped to almost all level 1 functions, with the exception of 6 level 1 GO terms that mapped to only ABE and BE level 2 or level 3 functions. In particular, structural molecule activity (GO:0005198) was uniquely enriched in BE and/or E terms. Bacteria-specific (B) functions appeared substantially at level 3 and mapping to the oldest sets of level 1 functions. Collectively, these findings support the late functional diversification of Eukarya and Bacteria. The noteworthy result however was the huge number of level 2 and 3 GO terms belonging to the ABE group, followed in number by the BE group, that were enriched in the universal and most ancient binding (GO:0005488), catalytic (GO:0003824) and transporter activity (GO:0005215) functions. Thus, an important vertical trace exists that goes from ABE to BE, to E and finally to B, which uniquely supports the reductive evolution of Archaea and Bacteria, in that order, and a remarkable and late functional toolkit expansion in Eukarya, that was intimated in previous studies [7, 17].

### Convergence in evolution of molecular functions

From a technical point of view, we accept that the relationship between functions in the GO database makes it harder to make evolutionary inferences from genomic abundance (*g*) and distribution index (*f*) when coupled with phylogenetic reconstruction. However, the method was successfully applied to other studies [7, 8, 17, 21]. Moreover, genomic abundance has several advantages over other character types (including presence/absence) and the topologies of resulting ToFs are less affected by HGT or recruitment [62]. In our case, a function that was transferred or recruited into a genome must be fixed before spreading in the genome with time [7, 8, 17, 21]. The resulting changes in genomic abundance of that function impact its ancestry depending on background abundance levels; higher levels will be less impacted than lower levels and will be less affected by HGT or other convergences. Examples of ancient functions of these kinds include catalytic activity and binding. In contrast, convergences of rare functions with low genomic abundance levels can impact the relative placement of a function in the timeline. Examples include protein tag and metallochaperone activity. Despite these caveats, BS values can be used as measure of the robutness to the effect of HGT and recruitment since these factors induce variability in pairwise comparisons of genomes [17]. In our analyses, phylogenetic trees were strongly supported at their base allowing more accurate evolutionary prediction of ancient molecular functions.

The homoplasy of phylogenetic characters is a good indicator of the evolutionary impact of HGT [63]. Homoplasy portrays phylogenetic conflicts that arise when character change does not fulfill ‘shared and derived’ nesting patterns of single origin in a phylogeny. We calculated the homoplasy index (*Hi*) of level 2 and level 3 GO terms of the DAG_mf_ when they were used as characters. Remarkably, GO terms shared by the three superkingdoms (the ABE group) showed higher *Hi* values than other Venn taxonomic groups, much higher than the E group (S4 Fig). This observation supports the ancient role of genetic exchange prior to the rise of lineages from the urancestor of life. Similarly high *Hi* patterns were observed with the BE, AB and AE groups, suggesting wide genetic exchange with the ancient stem line of descent. Moreover, and as expected, *Hi* appears to decrease with the age of GO terms. The generally low *Hi* values of superkingdom-specific GO terms, which appear late in the timeline, support a limited role of HGT during the diversification of the functions of superkingdoms. We note that because we have followed a method to reduce HGT effects by excluding terms associated with problematic taxa [64], *Hi* may also reflect the effects of many-to-many relationships between GO terms and processes of recruitment [17]. A number of relationships and processes of these kinds have been used to explain modern molecular functions and their mapping to gene products and associated terminal GO terms [25, 65]. For example, diversification of functions, gene duplication, generation of multidomain protein families, broad target of protein active sites, and recruitment result in new functions that can contribute to homoplasy [17]. Operationally, this may result in redundancies at GO levels, overlappings of character definitions, and loss of phylogenetic information in characters, which usually results in poorly resolved trees [66]. This appears not to be the case for our phylogenies.

### Early rise of metabolic activities through catalysis and binding

The ToF describing the evolution of level 1 GO terms revealed that the most ancient term at the highest level of the GO hierarchy is catalytic activity (GO:0003824), which is defined in the AmiGO database as: *“Catalysis of a biochemical reaction at physiological temperatures*. *In biological catalyzed reactions*, *the reactants are known as substrates*, *and the catalysts are naturally occurring macromolecular substances known as enzymes*, *Enzymes possess specific binding sites for substrates*, *and are usually composed wholly or largely of protein*, *but RNA that has catalytic activity (ribozyme) is often also regarded as enzymatic”*. The amino acid compositions of catalytic sites are evolutionarily conserved in protein enzymes [67, 68]. Similarly, their supporting structural domains are very ancient [69]. This is exemplified by the ancestrality and high conservation of the catalytic domains of aminoacyl-tRNA synthases, enzymes that are largely responsible for the specificity of the genetic code [54]. The ancient origin of catalysis provides a congruent evolutionary picture of structure and function that supports the “metabolism first” scenario of origin of life [70]. In this study we find that the appearance of catalytic activity occurred early and almost in parallel with binding function (GO:0005488), which is defined in the AmiGO database as: *“The selective*, *non-covalent*, *often stoichiometric*, *interaction of a molecule with one or more specific sites on another molecule”*. The importance of binding functions can be particularly seen at lower GO hierarchical levels, where they map to the most ancient GO terms at level 2 (GO:0043167–ion binding), level 3 (GO:0043168–anion binding) and terminal level (GO:0005524–ATP binding). Emergence of these functions is essential for the existence of small molecule metabolism, protein enzymes, and even translation (*see* Tables 3 and 4). These evolutionary patterns are remarkable and different from those observed in our previous study [17], probably because of the inclusion of archaeal taxa and a larger number of organisms (from 38 to 249) that are now providing a more balanced functional survey. Here we see that the most ancient functions at the lowest levels of the DAG_mf_ map to binding function. Once level 1 catalytic and binding functions were established, transporter activity (GO:0005215) appeared, which is defined in AmiGO as: *“Enabling the directed movement of substances (such as macromolecules*, *small molecules*, *ions) into*, *out of or within a cell*, *or between cells”*. We assume that the unfolding of catalytic machinery during this early period had to deal with the transport of larger molecules such as organic (GO:0097159), heterocyclic compounds (GO:1901363) and proteins (GO:0005515), as cell structure was unfolding. The level 2 ToF also revealed the early and explosive rise of metabolism that we previously inferred in a previous phylogenomic study of proteins [69]. The first enzymes were transferases (GO:0016740), followed by hydrolases (GO:0016787), oxidoreductases (GO:0016491), ligases (GO:0016829), lyases (GO:0016829) and isomerases (GO:0016853), in that order. This is in line with an analysis of protein domain recruitment in metabolic networks that showed that ancient enzymes were most likely transferases and hydrolases associated with nucleotide interconversion, storage and recycling of chemical energy through high energy phosphate transfer, and terminal production of nucleotides and cofactors [65, 69, 71, 72]. These molecular activities led to the inception of transmembrane transport (GO:0022857) of catalytic products, *“Enabling the transfer of a substance from one side of a membrane to the other”*.

The development of these very early metabolic activities was followed by a number of specific binding activities that would later enable primordial replication and translation mechanisms, including nucleoside phosphate binding (GO:1901265), nucleotide binding (GO:0000166), ribonucleotide binding (GO:0032553), and nucleoside binding (GO:0001882), which are part of the most ancient functions of the timeline of level 3 GO terms. Another important feature is the dependence of ancient molecules on small molecules such as ATP-binding and cofactor binding (GO:0048037), which are necessary for cellular energetics [69, 73]. Similarly, level 2 GO terms at the base of the ToF back up the early discovery of binding of small molecules and cofactors and late discovery of binding of single atoms and interactions with transition metals needed for more efficient energy dissipation in cells [17]. Moreover, early interaction of cofactors and enzymes and late involvement of transition metals were supported by a phylogenomic investigation of metal binding protein domains leading to coordinated chemistries typical of enzymes [21, 74]. This result agrees with transition metals, such as zinc, copper, iron, and manganese, being crucial for modern life as well as for the environment of primordial life. Metals are known to interact with enzymes involved in nucleic acid replication, transcripton, protein-protein, protein-DNA, and protein-RNA interaction (Zinc finger proteins), and also photosynthetic proteins. All of these processes require broadly defined binding functions.

Phylogenomic analysis revealed that ion binding functions (GO:0043167) at level 2 and anion binding functions (GO:0043168) at level 3 of the GO hierarchy were the oldest molecular functions at these levels. The primordial activity of level 1 binding activity (GO:0005488) to components of nucleic acid and protein macromolecules expressed at lower level GO terms, including level 2 heterocylic compound binding (GO:1901363), organic cyclic compound binding (GO:0097159), small molecule binding (GO:0036094) and cabohydrate derivative binding (GO:0097367). At level 3, anion binding was followed by nucleotide binding (GO:0000166) and nucleoside phosphate binding (GO:1901265). We interpret these primordial progressions as descriptions of a functional repertoire of molecules that was much simpler and less efficient than current repertoires, which had to operate with less diverse chemistries and under environmental conditions that are inexistent today [17]. While our interpretations are related to modern molecular functions, we also explain the rise of modern definitions of molecular functions as palimpsests of ancient chemistries operating already at planetary level. This assumption has been supported by recruitment patterns in metabolism [17, 71]. Note that the discovery of catalytic activity is embodied in more than half of all genes that were investigated and increased in number in Archaea, Bacteria and Eukarya, in that order (Fig 5A, 5B and 5C), which follows genome complexity and the evolutionary rise of superkingdoms. It is very interesting that this early function was massively adopted by organisms spreading into low levels of the GO hierarchy and during the entire evolutionary timeline. We also see the dominance of binding activity defined with level 2 GO terms (see Fig 15A) in the very early evolutionary timeline of molecular functions (*nd* < 0.4). During that time the diversity of the reportoire of level 2 GO terms corresponding to level 1 GO terms was considerable. We assume that ancient catalytic machinery diversified into several modern functions embodied in ontological definitions of catalytic, binding, and transporter activities. This process of diversification mirrored the massive diversification of protein fold structures during the evolution of the protein world [30].

### Biocatalytic mechanism and function

The development of catalytic mechanisms requires unfolding new stepwise chemical reaction mechanisms in related enzymes. When biocatalytic mechanisms were investigated, the P-loop containing nucleotide triphosphate hydrolase and the NAD(P)-binding Rossmann-like homologous superfamilies (the oldest two structures) introduced many mechanistic steps, which then combinatorially distributed in catalytic history [75]. In parallel to the mechanistic approach, our model proposes a metabolism first scenario of origin of life and hence, that the oldest enzymes supplied a sufficiently flexible scaffold to aid a number of mechanistic step types effecting their reactions [75]. Moreover, the primordial functional toolkits made use of inorganic chemistry and then of more complicated organic chemistry (see Tables 3 and 4). These trends require many recruitment events, including those associated with the hourglass pattern behavior of the functional network (Fig 16) and diversity of catalytic mechanisms in the discovery of EC functions [17, 75].

### Late rise of macromolecular biosynthesis

Molecular activities centered in proteins and nucleic acids arise soon after the rise of catalysis, binding and transport. The fifth GO term of the level 1 timeline, GO:0000988–protein binding transcription factor activity, is defined as: *“Interacting selectively and non-covalently with two or more protein molecules*, *or a protein and another macromolecule or complex*, *permitting those molecules to function in a coordinated way*, *in order to modulate transcription*. *A protein binding transcription factor may or may not also interact with the template nucleic acid (either DNA or RNA) as well*.*”* This high level term shows a change of focus from metabolism to primordial replication and translation mechanisms linked to the protein biosynthetic function [17, 62]. Primordial hydrolases (ATPase and GTPase) likely utilized the energy of nucleotide binding and hydrolysis to exert mechanical work [76], which at this stage involves polypeptides (level 2 GO:0005515–protein binding) and nucleic acids (level 2 GO:0000990–core RNA polymerase binding transcription factor activity). There are several level 3 functions that developed during this time, which facilitate binding of nucleic acids (GO:0003676), RNA polymerase modulation of transcription (GO:0000990), and a core DNA-dependent RNA polymerase binding to form a holoenzyme complex, and also, while present in the holoenzyme, interacting with promoter sequences in order to confer sequence specific promoter recognition (GO:0000996) (see definitions in AmiGO database). The relatively late development of translation is congruent with patterns of evolution of protein domain structure [30, 65]. Protein synthesis likely evolved from ligase activity of an ancient form of class II aminoacyl-tRNA synthetase (aaRS) (GO:0004812) [72]. Moreover, coevolution and accretion of protein domains and molecules resulted in modern aaRSs and non-ribosomal protein synthetases (linked to the quite early level 3 GO:0016875–ligase activity, forming carbon-oxygen bonds), and ribosomal constituents [17, 72]. This model, which is compatible with the results of our ToFs, provides explanation of how primordial functions are linked to structure and how their interactions with metals and cofactors developed into modern translation (for detail, *see* [72]). Following the emergence of universal GO terms at the base of the ToF, more specialized molecular functions for replication and translation appeared later in the evolutionary timeline that were not universally spread in functionomes. For example, sequence-specific DNA binding transcription factor activity (GO:0003700), transcription factor binding transcription factor activity (GO:0000989), DNA polymerase processivity factor activity (GO:0030337), translation regulator activity, nucleic acid binding (GO:0090079), DNA topoisomerase activity (GO:0003916), sequence-specific DNA binding RNA polymerase II transcription factor activity (GO:0000981), and RNA polymerase II transcription factor binding transcription factor activity (GO:0001076) proceeded in very defined order in the timeline of level 3 GO terms. These patterns of emergence of functions reflect a transition to more complex molecular machinery, high levels of processivity necessary for macromolecular biosynthesis and the operation of modern cells, the rise of DNA as repository of genetic information, and the rise of regulation at all levels of biological complexity [72].

### Interactions with the environment and the cellular self

The generation of ATP (the universal energy currency) from ADP, detoxification of xenobiotics by cytochrome P450, and other redox reactions led to the occurence of free radicals [77], which adversely affects the balance of metabolism and the functioning of living systems. Emerging cellular organisms had to adapt to different ocean and land enviroments and were forced to offset the effect of free radicals by generating protective mechanisms. This activity is reflected in the sixth GO term of the timeline of level 1 functions, antioxidant mechanism (GO:0016209), which is defined in AmiGO as: *“Inhibition of the reactions brought about by dioxygen (O*_*2*_*) or peroxides*. *Usually the antioxidant is effective because it can itself be more easily oxidized than the substance protected*. *The term is often applied to components that can trap free radicals*, *thereby breaking the chain reaction that normally leads to extensive biological damage*.” The activity manifest in the timeline of functions with the early appearance of level 2 GO terms such as peroxidase activity (GO:0004601), superoxide dismutase activity (GO:0004784), and thioredoxin-disulfide reductase activity (GO:0004791), and oxygen binding (GO:0019825), and level 3 GO terms such as catalase activity (GO:0004096), NADH peroxidase activity (GO:0016692), cytochrome-c peroxidase activity (GO:0004130), and NADPH-adrenodoxin reductase activity (GO:0015039). The number of interactions with the environment and the sensing of the cellular self unfold as the numbers of molecular functions expand in living systems. The progressive development of disposal of by-products (GO:0015643–toxic substance binding; GO:0019534–toxin transporter activity), sensing of pathogenic agents (GO:0001618–virus receptor activity), induction of immune system (GO:0003823–antigen binding), and control of cell death (GO:0002039–p53 binding) occurs in parallel with the diversification of organismal lineages. These developments, which can be traced in the timelines, show increasing interactions with biotic and abiotic entities and increased control of the internal cellular state. We assume that metabolic networks responded progressively to environmental change and big planetary transitions in the timeline. There are two obvious oxygen energy-linked subnetworks, oxygenic mitochondrial ATP synthesis and oxygenic photosynthesis, developing quite late in evolution, well after the discovery of most enzymatic activities [69]. Remarkably, the level 3 GO term catalase activity (GO:0004096) was the earliest function linked to planetary oxygenation. Its appearance (*nd* = 0.403) was not so distant from the appearance of the first BE–specific level 3 function (*nd* = 0.452), which signals the start of organismal diversification. The origin of catalase domains and the rise of planetary oxygen and aerobic metabolism have been recently traced in evolutionary timelines of protein structural domains [78]. Results suggest the catalase enzyme was crucial for planet oxygenation, triggering the rise of organismal lineages. This is compatible with molecular and geological records that assume life developed considerable complexity before the appearance of oxygen in the atmosphere [69, 78–80]. In fact, enzyme distributions in aerobic pathways suggest adaptation to oxygen occurred after major prokaryotic divergences in the tree of life.

### Cellular adaptation to planetary oxygen

Many vital cellular processes including photosynthesis, respiration, and a number of metabolic reactions involve electron transfer among proteins [81] and/or between protein and metabolites. Following the establishment of interactions with the environment and the cellular self, which adversely affects the balance of metabolism and the functioning of living systems, electron carrier activity (GO:0009055) is the seventh GO term to emerge in the timeline of level 1 molecular unctions. It is defined in AmiGO as: “*Any molecular entity that serves as an electron acceptor and electron donor in an electron transport chain*. *An electron transport chain is a process in which a series of electron carriers operate together to transfer electrons from donors to any of several different terminal electron acceptors to generate a transmembrane electrochemical gradient*”. The electron carrier activity also manifests in the early appearance of the level 2 GO term cytochrome-c oxidase activity (GO:0004129, *nd* = 0.32). Cytochrome-c oxidases catalyze the reduction of oxygen to water with production of ATP [82]. It is well accepted that atmospheric oxygen increased as a result of oxygenic photosynthesis. Although oxygen distributed in the Earth 2.3–2.4 Gyr ago during the Great Oxidation Event [83–85], the record of aerobic metabolism in the chronology of F and FSF indicates that aerobic respiration occurred 2.8 Gyr ago and that the most ancient aerobic biosynthesis happened 2.9 Gyr ago [78, 79]. The relatively early development of aerobic metabolism is compatible with patterns of evolution of protein domain structure. The early appearance of cytochrome-c oxidase could be related to the emergence of an ancestral oxidase (uroxidase) [82]. Probably, small amounts of oxygen could have been formed as a result of water photolysis, leading to the possible availability of “oxygen oases” in localized region of the ocean surface [86]. The organisms having the uroxidase could have lived in the restricted environments, which facilitated the development of a primordial aerobic metabolism [82]. Of course, oxygen provides benefits to the evolutionary progression such as birth of eukaryotes and new reactions discovering novel metabolites [78]. Remarkably, the appearance of GO:0004129 was not so distant from the appearance of the first Eukarya-specific level 2 functions. Thus, the electron carrier activity GO:0004129 term jump-started oxygen adaptation before the diversification of major prokaryotic lineages.

### Enzyme regulation

Other enzymes can control enzymatic activity. Similarly, enzyme levels can also be controlled. These modulations ensure proper pathways/metabolite output necessary to meet the biological demands of the cell. These control mechanisms correspond to the enzyme regulator activity (GO:0030234) level 1 GO term, defined as “*Binds to and modulates the activity of an enzyme*”, which is the eighth level 1 term to appear in the timeline. Its appearance represents a change of focus from metabolism to flux control of pathway/metabolites. For example, nucleoside-triphosphatase regulator activity (GO:0060589, *nd* = 0.407) modulates the rate of nucleoside triphosphate (NTP) hydrolysis by NTPase, avoiding ATP waste. Similarly, kinase regulator activity (GO:0019207, *nd* = 0.458) involves the modulation of kinases that transfer a phosphate group usually from ATP. Phosphorylation or dephosphorylation processes play crucial roles in regulating the activity of proteins and in the processing of extracellular signals in eukaryotic cells [87]. In general, metabolic input-output relationships can turn on-off entire pathways, which means enzymes operating at the time when the cell requires a biochemical reaction to occur. Remarkably, the regulation of the activity of proteins in regulatory and signalling pathways unfolded in the 0.4 <*nd* ≤ 1 age range after the inception of GO:0030234. It is apparent that enzyme regulator activity was introduced into functionomes only after development of several important blanket molecular activities, including catalytic activity, macromolecular biosynthesis, and antioxidant activity. This signifies a new era for organismal lineages with more balanced and programmable metabolic networks.

### The rise of structural and cellular complexity

When we focus on structural molecular activity (GO:0005198), the ninth level 1 GO term of the timeline, the term illustrates the action of molecules associated with integrity of a complex or a structure in a cell or living organisms. We note that our dataset does not contain GO:0003735–structural constituent of ribosome based on our selection criteria since it is enriched by HGT [21]. Thus, we cannot speculate on the appearance of ribosomal structures. However, Caetano-Anollés et al. [72] suggested that the early presence of aaRSs, and then aaRs-transcription factor-nucleic acid minihelix complexes, faciliated the development of the emerging function of protein synthesis leading to the ribosome complex. The timeline of level 2 molecular functions revealed the progression of structural constituent of the eye lens (GO:0005212), muscle (GO:0008307), myelin sheath of vertabrate nerve (GO:0019911), extracellular matrix (GO:0005201), nuclear pore (GO:0017056), egg coat (GO:0035804), cytoskeleton (GO:0005200), cell wall (GO:0005199), chorion (GO:0005213), and protein complex scaffold (GO:0032947). This progression uncovered a gradual increase of cellular complexity in evolution and milestone structures such as the nuclear pore of the eukaryotic cell and the complex structure of the eye. It also showed that subsets of eukaryal specific functions appeared earlier than bacterial counterparts [17].

The emergence of the two main types of glycerol membrane lipids (*sn1*,*2* fatty acid ester and *sn2*,*3* isoprenoid) is necessary to understand organismal adaptation to high temperature and the origin of the Archaea [88–90]. For the reduction of the keto group in dihydroxy acetone phosphate, a glycerol-3-phosphate dehydrogenase (GO:0004368 linked to GO:0016614–oxidoreductase activity, acting on CH-OH group of donors) is present in Bacteria and Eukarya, and a glycerol-1-phosphate dehydrogenase (GO:0050492 linked to GO:0016614) in Archaea [90]. Phylogenomic analysis of molecular functions shows thermophilic Archaea emerged earlier than the other two superkingdoms [21]. Moreover, archaeal microbes are characterized by ether-isoprenoid lipids, which are adapted to high temperature and other stress conditions [21, 90]. Conversely, Eukarya has double layer membranes of ester-fatty acid lipids since eukaryal organisms are adapted to moderate temperature. Therefore, either ester or ether, a primary seperation exists between the two kinds of cells: on one side Archaea (*sn2*,*3*) and on the other side Bacteria and Eukarya (*sn1*,*2*) [90, 91]. The phospholipid constituiton of modern membranes developed gradually in evolution, since GO:0005543–phospholipid binding appeared later at level 3. On the other hand many peptides that are integral to membranes would not only explain the origin of modern transport (e.g., GO:0005515–protein binding, GO:0022892–substrate-specific transporter activity) and channel forming activity but also enable the selective binding of metals, cofactors (GO:0048037), nucleic acid, protein complexes, and other chemicals in protocells with a membrane structure similar to that of modern organisms [72].

### A consistent historical narrative of functional origins and molecular evolution

Our study of functional origins dissects in iterative manner the most ancient level 1 GO terms by identifying the most ancient functions at lower levels of the DAG_mf_ hierarchy. Our analyses support the ‘metabolism-first’ view of origins of life, reinforcing the primacy of protein enzymes and ligand binding during early stages of cellular evolution. Results are consistent with previous findings derived from protein structure and function [7, 8, 21, 41, 42, 44, 46]. At the highest and ontologically broadest level of the hierarchy, the tree revealed that catalytic activity was the most primordial function, followed closely by binding (Fig 1). Catalytic activity was the most populated and diversified function at both level 3 and terminal level (Fig 15). One assumption for origins of biochemistry is that promiscuous catalytic activities of emerging proteins provided an initial selective advantage and with time accumulated and diversified to perform new metabolic functions [14, 36, 69]. This assumption is consistent with information in the evolutionary timeline of molecular functions. The ancient binding function contained the largest number of level 2 GO terms at *nd* < 0.4 and 0.6 ≤ *nd* ≤ 1 (Fig 15). Its basal placement suggests the crucial role of binding of compounds of many kinds in emerging catalytic sites of polypeptides. This is exemplified by binding of compounds important for central metabolic and informational processes, such as anion and cation binding (GO:0043168 and GO:0043169), carbohydrate derivative binding (GO:0097367), organic cyclic compound binding (GO:0097159), which are necessary for genetics and protein synthesis (*see* Tables 3 and 4). The universal presence of these functions (*f* = 1) support its central role and suggests that the uranancestor was particularly enriched with metabolic functions [7] and an emerging macromolecular biosynthetic machinery [17]. In fact, proteins are known to bind to other molecules such as anions, cofactors, vitamins and more complex molecules. However, protein binding always displays great specificity, in the sense that each protein molecule can usually bind just one or a few molecules out of the many thousands of different types it encounters [92]. The catalytic activity of enzymes converts bound molecules into metabolic components. Phylogenomic analysis of domain structures showed that the most ancient catalytic activities were embedded in P-loop hydrolase (c.37) fold structures harboring transferase, hydrolase, lyase, ligase and oxireductase catalytic activities [69]. All of these functions were consistently ancient as life evolved at different levels of structural classification. This pattern matches the ancestries of these enzymatic functions in ToFs (Fig 7). We find that transferase and hydrolase functions were the most ancient enzymatic activities, though in reverse order when compared to a previous study [17]. Transferases are involved in metabolic network formation by transferring groups of molecules from one metabolite to another, a process that requires transport activities (GO:0022892–substrate-specific transporter activity). Pfeiffer et al. [93] suggested that transferases were crucial for the emergence of hub metabolites and specialized enzymes originating from multifunctional enzymes. This is also congruent with the assumption that the most ancient functions are catalytic and pluralistic. As ToFs travel deeper into more specific terms, the basal taxa were followed by enzymatic activies in level 2 and level 3 GO terms, including transferring phosphorus-containing groups (GO:0016772, EC 2.7), peptidase activity (GO:0008233, EC 3.4), hydrolase activity, acting on acid anhydrides (GO:0016817, EC 3.6), hydrolase activity, acting on ester bonds (GO:0016788, EC:3.1) (*see* Tables 3 and 4) as broad candidates for an origin of metabolic networks in nucleotide metabolism [17, 69]. Following the origin of metabolic functions, our historical narrative of molecular evolution reveals the evolutionary development of functional toolkits of macromolecular biosynthesis, controlled interactions with the environment and self, adaptation to oxygen, enzyme regulation in coordinated networks and finally the rise of structural and cellular complexity. Remarkably, evolution unfolds in ways that are consistent with the biological and geological records, major evolutionary transitions, and the expected complexification of the molecular and cellular world.

We end by noting that the GO database is organized around a set of functions with widely differing granularities, while also putting together entities and concepts that often pertain to widely different functional fields [18, 38–40]. The effort started with the analysis of three model organisms (fly, mouse and yeast) and is now being extended to all domains of life. Necessarily, the growth of the GO database as a tangle-like and complex structure is constrained by knowledge acquisition, which exhibits highly heterogeneous impetus across scientific fields (see the ‘Benefits and disclaimers’ section of Materials and Methods). This impacts the breath of the taxa being studied and the functionomes that are being sampled in the effort to dissect the history of molecular functions.

## Materials and methods

### Data

We recently exploited GO terms in both a non-historical and a historical phylogenomic study to describe the evolution of modern cells [8, 21, 41, 42, 46]. Here, we reused the dataset after removal of HGT-prone genes to confirm the inferences drawn in Nasir et al. [21] by conducting specific analyses on the origin and spread of GO_TMF_ terms in the functional hierarchy. Gene Ontology Association (GOA) files of completely sequenced proteomes were downloaded from the European Bioinformatics Institute (EBI, November 2009). For confirmation, we scanned 1924 GO_TMF_ terms for the update (October, 2014). This resulted in a repertoire of 1,891 GO_TMF_ terms in 249 organisms, including 45 Archaea, 183 Bacteria, and 21 Eukarya with free-living lifestyles.

We began our initial phylogenetic analyses by assigning GO_TMF_ terms to corresponding levels 1, 2 and 3 of GO classification by using the Gene Ontology Online SQL Environment Tool (http://www.berkeleybop.org/goose). Level 1 terms are directly linked to the root GO term- molecular function. Level 2 terms are child nodes of level 1 GO terms. Although the AmiGO browser provides 21, 180 and 742 GO terms at levels 1, 2, and 3 respectively (October, 2014), we filtered GO_TMF_ terms based on the following four criteria: (i) parent GO_TMF_ terms with no children were considered as taxa in phylogenetic analyses at the child level; (ii) all taxa at the same level must exhibit an ‘is-a’ relationships with their parent GO_MF_ terms. If a GO_TMF_ term has a ‘part_of’ or ‘regulation’ relationships with its parent, the term was removed; (iii) GO_TMF_ terms with multiple parental nodes that appeared frequently at a certain level were treated as individual taxa; and (iv) GO_TMF_ terms positioned at different levels of the DAG were not excluded. We then proceeded to reconstruction phylogenetic trees from genomic abundance of GO terms per taxon for each of the 249 genomes that were analyzed.

### Phylogenetic analyses of function at high hierarchical levels

We used previously described methodology to generate ToFs and ToLs portraying the evolution of GO_TMF_ terms and species, respectively [8, 13, 17, 21]. We first calculated the number of times each GO_MF_ term was present in every genome and constructed a matrix representing the census of molecular functions in genomes. Here, the number of terminal GO terms per taxon per genome was defined as genomic abundance (*g*). The raw counts of the genomic abundance of each GO_MF_ term in every genome (*g*_ab_) were log-transformed to account for unequal genome sizes and heterogeneous variances and then divided by the maximum abundance value (*g*_max_) in the matrix. The equation below describes the data manipulation procedure.

$$g_{\mathrm{ab\_norm}}=\mathrm{Round}\;\left[\ln\left(g_{ab}+1\right)/ln(g_{\max}+1)*31\right]$$

Using this method, the genomic abundance value for GO terms in every functionome (*g*_ab_) was standardized by the maximum value in the matrix (*g*_max_) and normalized to a scale from 0 to 31. The standardized counts were then rescaled using an alphanumeric format (0–9 and A–V) with 32 possible character states to allow compatibility with PAUP* phylogenetic reconstruction software (ver. 4.0b10) [94]. Furthermore, normalization and rescaling of raw abundance values into 32 possible character states (0–9 and A–V) reduce the likelihood of convergent evolution. Maximum parsimony (MP) was used to obtain the most parsimonious tree describing the evolution of GO terms with minimum possible character changes. Unrooted most parsimonious trees were generated with heuristic searches, using stepwise addition and tree bisection and reconnection (TBR) as the branch swapping algorithm. Trees were polarized *a posteriori* with the ANCSTATES command in PAUP*. Since optimization used Wagner ordered characters, character polarization can be accomplished in only two possible directions, one of which results in character change that is nested in the branches of the rooted trees and complies with Weston’s rule. For ToLs, 0 was initially specified as the ancestral character state; character state polarization assumed that ancient functionomes encoded only a handful of functions and progressively enriched their repertoires along the evolutionary timeline [17]. For ToFs, the maximum state was initially specified as the ancestral character state; character state polarization assumed that GO terms with largest abundance were the oldest as they had more time to accumulate functional variants in functionomes. Weston’s generality criterion was implemented with the Lundberg method that places the root at the most parsimonious location without any outgroup taxa specification [95]. Reconstructions confirmed the initial process-delimited polarization schemes. Homoplasy indexes (*H*_*i*_) were calculated for every character (level 1, level 2 and level 3 GO terms) with the PAUP option of ‘‘DIAG” (character diagnostics). Moreover, the phylogenetic error (i.e., effect of non-vertical evolutionary processes such as HGT and/or convergent evolution) was estimated by calculating retention indexes (RI) for GO terms. The RI indicates fit of characters to the phylogeny and is evaluated on a scale from 0 to 1 [96]. Higher RI values indicate better fit of phylogenetic characters and thus lower probability of non-vertical inheritance. The reliability of the phylogenetic trees was evaluated with 1,000 non-parametric bootstrap replicates and given as bootstrap support (BS) values for interior branches in a scale from 0 to 100%. BS values test the reliability of tree topologies by randomly resampling characters, building trees from the subsets, comparing topologies and determining the proportion of interior branches that are consistent with the original tree.

To measure the degree of monophyly of individual GO terms on a phylogenetic tree, we calculated the genealogical sorting index (GSI) using the GSI website (http://www.genealogicalsorting.org/) with 10,000 permutated replicates [97]. Trees were visualized using TreeGraph 2 [98].

### Estimating the origin of GO terms

We calculated the distance of each taxon (i.e., level 1 GO_MF_ terms) to the base of the rooted ToF using a PERL script that counts the number of nodes from a given position to the base and divides it by the total number of taxa. This node distance (*nd*) disclosed the relative age of each GO_MF_ term on a scale from most ancient (0) to most recent (1). The *nd* value has been successfully used previously in the evolutionary study of protein domain structure [30] and ontological data [8, 17, 21].

### Popularity of GO terms in genomes

We studied the spread of level 1, level 2, and level 3 GO terms in genomes. We utilized a distribution index (*f* value) to quantify the popularity of molecular functions. This index was calculated by dividing the number of functionomes (genomes) encoding a particular GO term by the total number of functionomes, on a scale from 0 to 1. Thus, an *f* value of 0 indicates complete absence of a GO term whereas a value close to 1 indicates near universal presence. Molecular activities that are crucial to cellular life were expected to have higher *f* values, while GO_TMF_ terms unique to a species or superkingdom were anticipated to have lower *f* values.

### Benefits and disclaimers

The use of GO terms for phylogenetic reconstructions carries both advantages and disadvantages when compared to molecular sequences and other molecular characters [8, 17, 21], some of which are listed in this section. Note that characters and taxa defining phylogenetic hypotheses are permanently revised. Our phylogenomic analyses attempt to describe a finite space of molecular functons (taxa in ToFs or characters in ToLs) and an ever growing space of functionomes (characters in ToFs or taxa in ToLs). While this manuscript focuses only on ToFs, the overall effort implies increasing the precision of the definition of characters and taxa within the confines of improvements, breath and growth of GO annotations of biological knowledge [38–40]. At present, the focus is restricted to the reconstruction of phylogenetic trees, with an understanding that with more computational power it would be possible to build other more informative evolution structures from phylogenetic character change information and search methods of tree optimization, including phylogenetic networks that explore multiple origins.

#### Advantages

(i) GO phylogenetic characters are collectively more conserved than sequence sites and less prone to change by site substitutions, though single mutations in a sequence (e.g. Interpro Q8IV63, inactive human serine/threonine-protein kinase VRK3 that cannot bind ATP) or a truncation of a domain (e.g. Interpro Q496M5, inactive human serine/threonine-protein kinase PLK5 lacking its autophosphorilation site) can inactivate a gene and eliminate/modify its associated GO terms, (ii) GO characters embody both deep and shallow history (from widely distributed in genomes such as ATP binding to more restricted such as diphosphokinase activity) while sequences can only dissect closely related relationships (e.g. [8]); (iii) GO characters benefit from functional conservation when non-homologous genes share a same functionality; (iv) GO characters suffer minimally from the effects of horizontal transfer, which notoriously complicate traditional sequence analysis [8], since HGT-prone genes were removed from raw datasets; (v) GO characters collectively portray the physiology of organisms while sequence sites and other characters describe individual molecules; and (vi) GO characters are highly resistant to violation of character independence when building ToFs since molecular functions in different organism seldom interact; in contrast molecular structure-induced site dependencies violate sequence site independence.

#### Disadvantages

(i) GO characters violate character independence when used to build ToLs [8,21] in cases where a molecular function is consequence of another (i.e. are co-occurrent); (ii) Despite limited effects on tree building and interpretations [8], evidence codes of genome annotations are biased, with most annotations being automatic predictions (~99%) that are computationally derived (e.g. homology, homeomorphy at sequence and structure level), including ‘inferred from electronic annotation’ (IEA) or ‘inferred from sequence similarity’ (ISS), and annotations associated with experimental evidence and manual curation are substantially enriched in model organisms [39]; and (iii) Since GO characters are based on GO annotations associated with terminal terms for genomes of different superkingdoms that are revised and with coverage that can change over time [40], it is possible that few terms sampled in this analysis will be later classified as parent terms for some other terms. Therefore, we caution the reader that our phylogenies and interpretations rest on GO definitions and relationships that are available at the time of analysis. However, while some details are expected to change, general trends and most patterns described in our study will likely remain unaffected with an increase in genomic data and GO community enhancements (e.g. for heart, kidney, development and apopotosis), refinements and revisions (e.g. improvement of terminology, identification of rare unsuitable direct annotations) to terms and relationships [40].

#### Other considerations

(i) Our study includes only GO terms of gene products that associate with a terminal GO term. Parent terms are then investigated at higher levels of the DAF_mf_. This diminishes biases arising from partial or incomplete annotations by focusing on the most specific and detailed ontological terminology of the most basal hierarchy and builds evolutionary depth bottom-up. To illustrate, UniprotKB A0A059TSS4 is associated with molecular function GO:0003700–transcription factor activity, sequence-specific DNA binding, which is a level 2 GO term, not a terminal term. The entry is excluded. Thus, we distinguish ‘association’ (links between gene products and the terminal GO terms) from ‘annotation’ (links among GO terms at different hierarchical levels). (ii) Molecular functions often spread by recruitment, manisfesting in unrelated metabolic enzymes with conserved catalytic triads and different GO terms. For example, aspartyl dipeptidase and LD-carboxypeptidase have a same catalytic triad but have GO:0008236–serine-type peptidase activity and GO:0004180–carboxypeptidase activity annotations, respectively [99, 100]. Both are unified by a same parent of DAG_mf_ at the highest level. In this regard, the difficulty of evolving novel stepwise chemical reaction mechanisms could be the dominant factor limiting the divergent evolution of new catalytic functions in related enzymes [75]. For example, Carrigan et al. [101] found that ancient human ADH4 (alcohol dehydrogenase class IV) enzymes could have not metabolized ethanol. However, enzymes did oxidize other alcohols, including terpenoid alcohols such as geraniol. A single mutation occurring ∼10 million years ago that endowed our ancestors with a markedly enhanced ethanol metabolizing ability. Conversely, a mutation could change the ability of catalytic activity of ancient enzymes resulting in inactive extant proteins. (iii) While automatic predictions are overwhelming, their accuracy and power is significant and subject to permanent quality assurance (e.g. applying taxon constraints to ensure term definitions are taxon neutral or the subject of post-processing) [40].

## Supporting information

S1 Fig Boxplot displaying the distribution of level 3 GO terms with respect to age (*nd*) in GO_TMF_ terms of 249 organisms. (TIF)

S2 Fig As an example, the figure displays several parents of one terminal GO term at different levels and also how one parent GO terms can appear at different levels at the same time. (TIF)

S3 Fig Bars illustrating the number of GO terms for level 1 GO category. (A) A total of 101 level 2 GO terms mapped to level 1 GO terms. (B) A total of 257 level 3 GO terms mapped to level 1 GO terms.
(TIF)

S4 Fig Character homoplasy index (*Hi*) of level 2 GO terms and level 3 GO terms. (A) On the phylogenetic tree of 249 genomes (TL = 7,731; CI = 0.284; RI = 0.685), *Hi* values for 101 level 2 GO terms that are parsimony-informative were calculated and plotted against *nd* values of GO terms derived from the tree. (B) On the phylogenetic tree of 249 genomes (TL = 21,461; CI = 0.267; RI = 0.649), *Hi* values for 257 level 3 GO terms that are parsimony-informative were calculated and plotted against *nd* values of GO terms derived from the tree. Colored circles denote GO terms that are present in superkingdoms. Quartile values (Q1 and Q3) were determined from the distribution of *Hi* values of GO terms.
(TIF)
