# Supplementary figures and images for: The natural history of molecular functions inferred from an extensive phylogenomic analysis of gene ontology data
Source: PLoS One. 2017 May 3;12(5):e0176129. doi: 10.1371/journal.pone.0176129 (PMC5414959; doi:10.1371/journal.pone.0176129)

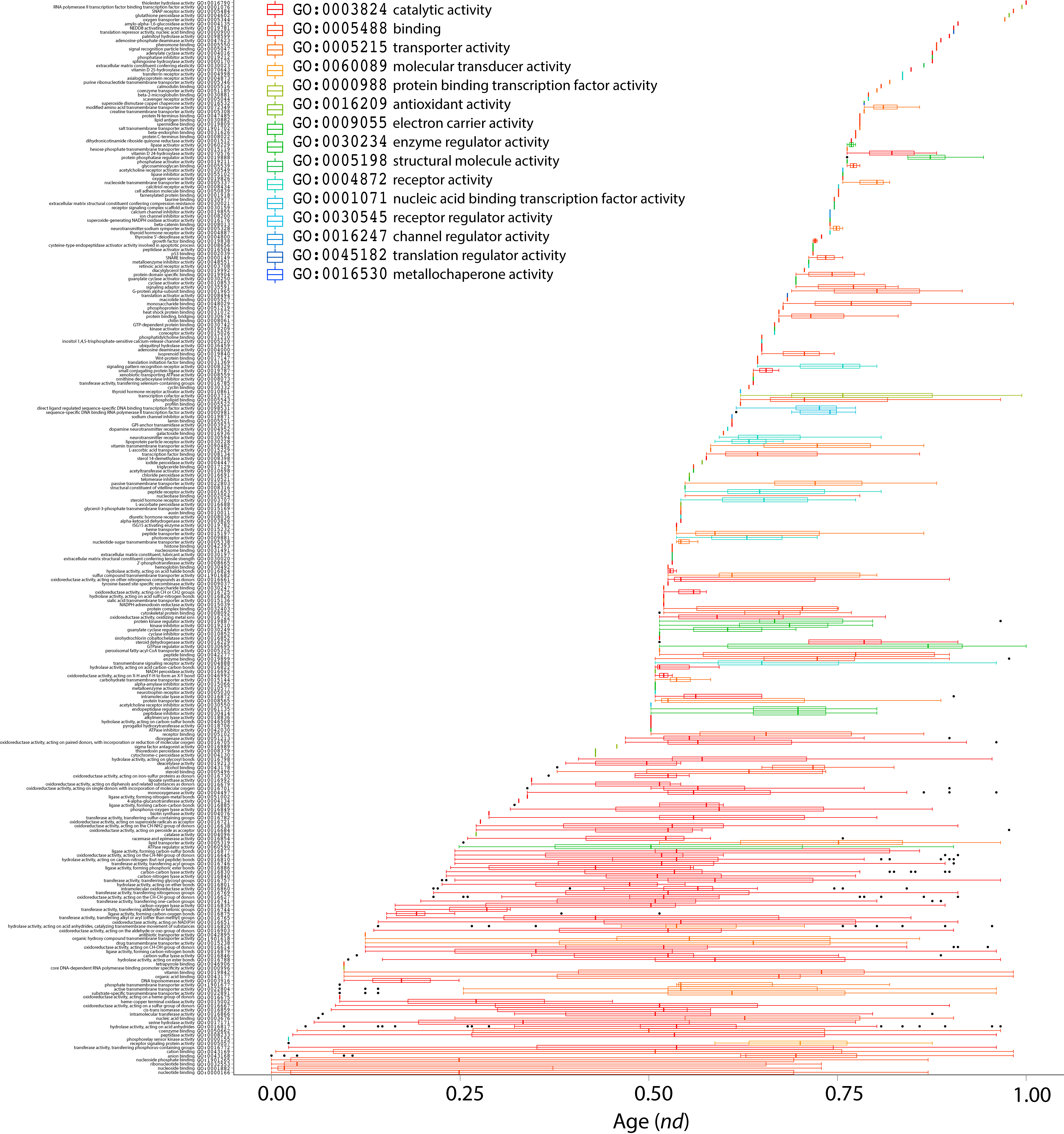

Supplement: S1 Fig — (TIF) [file pone.0176129.s001.tif]

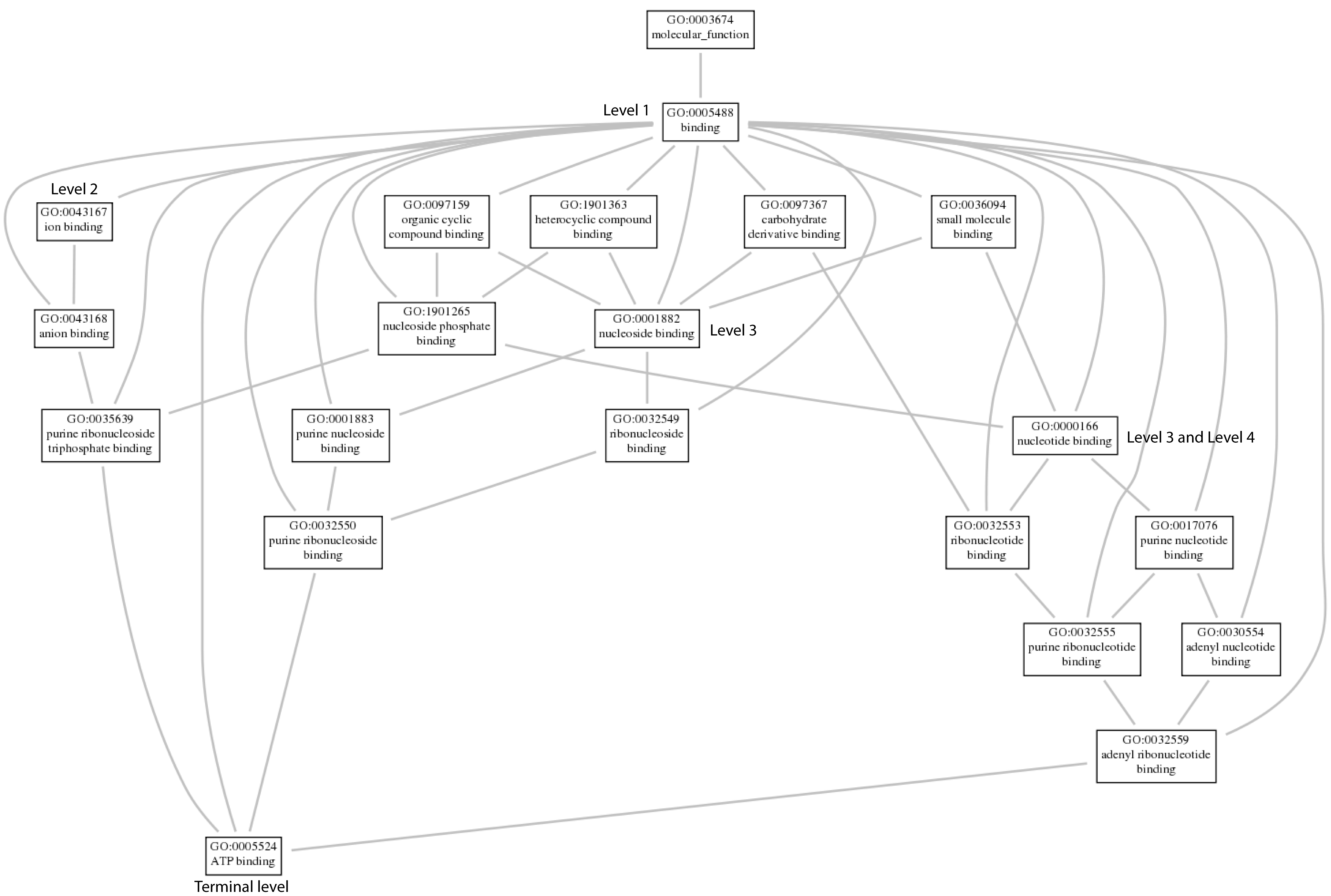

Supplement: S2 Fig — (TIF) [file pone.0176129.s002.tif]

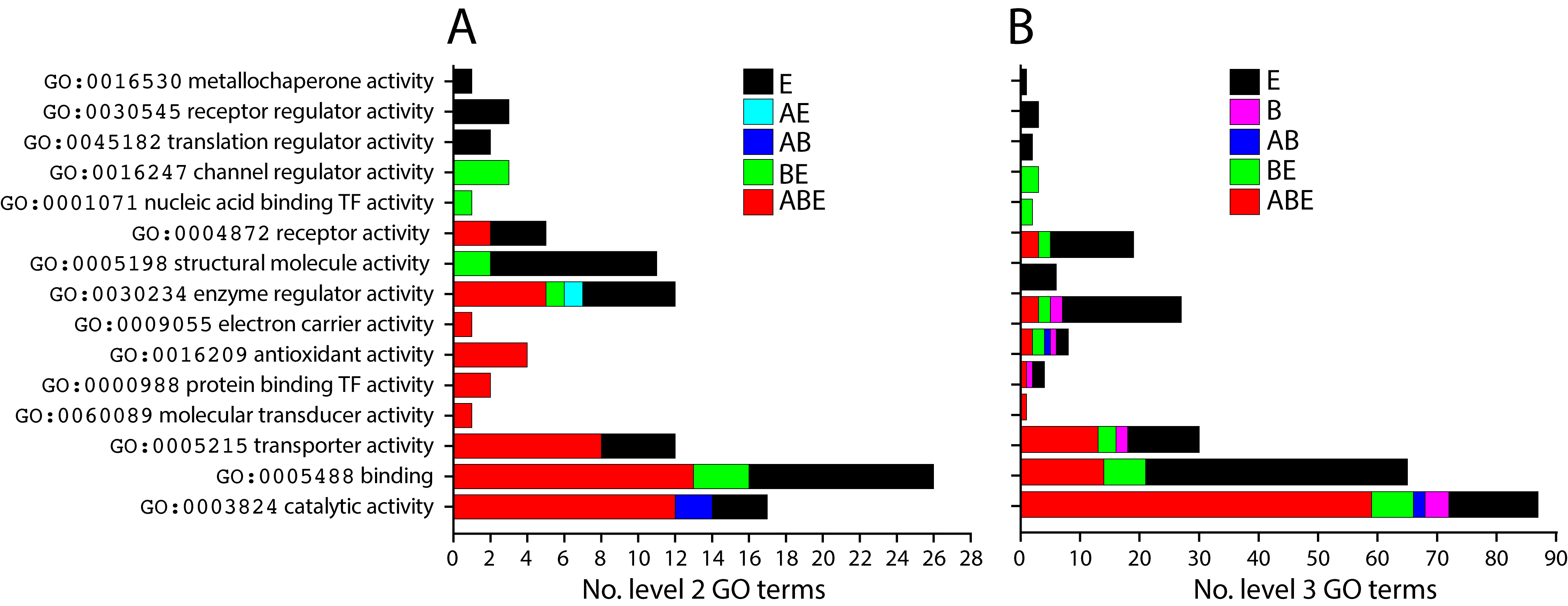

Supplement: S3 Fig — (A) A total of 101 level 2 GO terms mapped to level 1 GO terms. (B) A total of 257 level 3 GO terms mapped to level 1 GO terms. (TIF) [file pone.0176129.s003.tif]

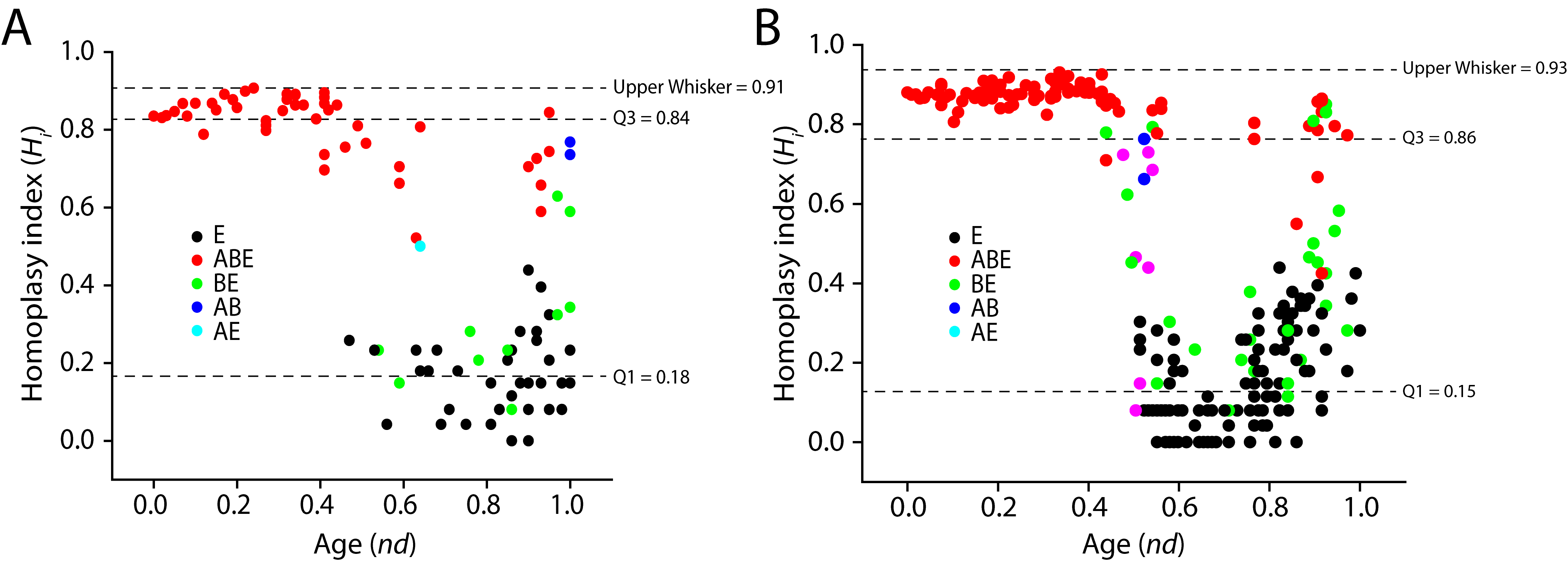

Supplement: S4 Fig — (A) On the phylogenetic tree of 249 genomes (TL = 7,731; CI = 0.284; RI = 0.685), Hi values for 101 level 2 GO terms that are parsimony-informative were calculated and plotted against nd values of GO terms derived from the tree. (B) On the phylogenetic tree of 249 genomes (TL = 21,461; CI = 0.267; RI = 0.649), Hi values for 257 level 3 GO terms that are parsimony-informative were calculated and plotted against nd values of GO terms derived from the tree. Colored circles denote GO terms that are present in superkingdoms. Quartile values (Q1 and Q3) were determined from the distribution of Hi values of GO terms. (TIF) [file pone.0176129.s004.tif]
